# Effect of Drying Time and Frying Conditions on the Quality of Pork Rinds by Response Surface Methodology

**DOI:** 10.1002/fsn3.4513

**Published:** 2024-11-01

**Authors:** Shang‐Ta Wang, Hong‐Ting Victor Lin, Yu‐Jin Syu, Wen‐Chieh Sung

**Affiliations:** ^1^ Department of Food Science National Taiwan Ocean University Keelung Taiwan, Republic of China; ^2^ Institute of Food Safety and Risk Management National Taiwan Ocean University Keelung Taiwan, Republic of China; ^3^ Center of Excellence for the Oceans National Taiwan Ocean University Keelung Taiwan, Republic of China

**Keywords:** deep‐fat frying, pork rind, process optimization, response surface methodology

## Abstract

Fried pork rind, a processed pork by‐product, is popular as a snack globally, prized for its distinctive flavor and crisp texture achieved through frying. Although various studies have examined processing factors such as thickness, moisture content, and brine concentration, there is a scarcity of research addressing the effect of frying temperature on the quality of fried pork rinds. In the present study, the effects of varying hot air drying times (12, 18, and 24 h at 50°C), traditional deep‐fat frying temperatures (180°C, 195°C, and 210°C), and frying durations (3, 4, and 5 min) on the oil content, moisture content, breaking force, color, puffing ratio, and microstructural appearance of pork rinds were evaluated. The results revealed a significant correlation between frying temperature and time with the oil content of the pork rinds. The oil content and puffing ratio peaked at approximately 195°C. Moreover, the breaking force of the pork rinds decreased with increased frying time at 180°C, while the opposite trend was observed at 210°C.

## Graphical abstract

Correlations between frying conditions and the quality of pork rinds were identified. Correlations between pretreatment conditions and the quality of pork rinds were revealed. The optimal processing conditions for fried pork rinds were predicted and verified.

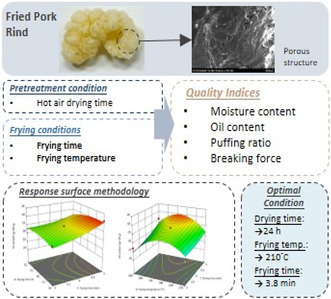

## 1 Introduction

Pork (*Sus scrofa domestica*) rinds, with a protein content of 36.1%, have been fried and used as ingredients in side dishes and puffed snack foods, offering a high‐protein option with a unique flavor and crispy texture (Villar 1994; de Oliveira Fagundes et al. 2017). Rind comprises 4%–6% of the total carcass weight of pork (Boler 2014). Frying is a complicated process in which food undergoes several physicochemical reactions, including oxidation, glass transition, polymerization, hydrogenation, and puffing. During this process, food absorbs oil or fat and loses water, while also experiencing protein denaturation, nonenzymatic browning, and starch gelatinization (Mittal 2009; Jothi et al. 2018). Traditional frying, usually at 180°C, can form diverse aromas called reaction flavors, giving rich flavors to fried food (Oladejo et al. 2018). Potato chips and French fries are the commonly studied fried snacks and food. Pork rind, fried in oil at 200°C–220°C to achieve the desired texture and seasoned with salt, is sold as a snack food in various Asian countries including Vietnam, the Philippines, Taiwan, and Thailand, as well as in the United States (Serna‐Saldivar 2022). However, fried pork rind contains 30%–40% oil/fat, making it an unhealthy but popular snack (USDA 2021). Eating fried food is usually associated with negative concerns, including fat‐related coronary heart disease, obesity, and diabetes (Cahill et al. 2014; Cai et al. 2012), by consumers. Food research communities, manufacturers, and consumers are interested in low‐oil‐absorption snacks that maintain the desirable organoleptic characteristics of fried foods.

Various thicknesses (2, 3, and 4 mm) and frying times (0.5–5 min) after drying were evaluated in traditional deep‐fried snacks. The results indicated that the puffing ratio, breaking force, and oil content of pork rind did not significantly differ across the tested thicknesses when fried for 3–5 min at 180°C, with water content ranging from 1.68 to 1.98 g/100 g on a wet weight basis (Lin, Hou, and Sung 2021). According to sensory panelists, fried pork rinds with a thickness of 4 mm were rated lower in texture, flavor, and overall acceptance compared to those with thicknesses of 2 and 3 mm. Irregular and fewer holes and crack microstructures on the fried pork rind surface were observed under a scanning electron microscope compared with raw rind samples with a thickness of 4 mm.

In our previous study on fried pork rind (Lin, Hou, and Sung 2021), we found that raw pork rind thicker than 4 mm should be fried at temperatures exceeding 180°C to achieve good sensory acceptability and puffing ratio. However, due to methodological limitations in that study, the precise optimal conditions were not clearly determined. We proposed that response surface methodology (RSM) could address this issue. RSM is a valuable technique for designing, formulating, and analyzing products, enabling fewer experimental trials and more rapid identification of optimal product quality in the food industry. Additionally, the water content of the semidried product before frying is a critical factor influencing the puffing, texture, and overall puffing ratio of the fried product (Jia et al. 2018). Furthermore, drying cooked pork rind to form transparent gels before traditional deep‐frying puffing at 190°C could be a critical factor in optimizing the puffing ratio (Truong et al. 2014).

This study aimed to evaluate the physical properties of fried pork rind under various drying times, frying temperatures, and frying durations using RSM. The objective was to optimize the drying time, frying time, and temperature, to achieve a reduction in oil uptake, breaking force, and the maximum puffing ratio of the pork rind.

## 2 Materials and Methods

### 2.1 Raw Materials and Dried Pork Rind Processing

Frozen pig (*Sus scrofa domestica*) rind was obtained from Warehouse Full Meat Product Enterprise Co., Ltd. (Hsinchu City, Taiwan). For this study, the pork skins were defrosted under running tap water, sorted by thickness, and then cooked for 1 h in boiling water. The subcutaneous rind fat layer was removed, and the pork rind (average thickness: 3.7 ± 0.3 mm) was tailed into 2 × 5 cm and dried for 12, 18, and 24 h at 50°C. Dried pork rinds were kept in polythene bags until the time of frying.

### 2.2 Deep Frying and Production of Pork Rinds

Dried pork rinds (10 pieces) were fried in a fry pan (5 L) with refined palm olein oil (2.5 L). The temperature in degrees of Celsius of frying oil was read using a digital wired probe thermometer (Model WG‐79, Truepot Trading Co., Ltd., Taipei City, Taiwan). Dehydrated pork rinds were fried at 180 ± 5°C, 195 ± 5°C, and 210 ± 5°C for 3, 4, and 5 min. During frying, the pork rinds were fried in a fry pan, and the pork rinds were collected from the fryer and centrifugalized in a de‐oiler (Model DOO‐130, Yu Sheng Guang Food Machine, Taichung, Taiwan) at 236 rpm for 1 min for de‐oiling. The equipment measures 38 × 28 × 37 cm, which is well‐suited for the batch quantities required in our experiment. The frying oil was changed after four frying sample batches in 2 h.

### 2.3 Lipid and Water Content and Water Activity

The lipid content of pork rinds was evaluated by diethyl ether extraction (Association of Official Analytical Communities, AOAC 2000). The fried pork rind lipid content was measured gravimetrically by lipid extraction using a Tecator SoxtecSystem HT‐1043 (Foss Analytical Co., Ltd., Hillerod, Denmark). Water content was tested according to AOAC 984.25 (AOAC 2000). The water activity of pork rinds was tested by a water activity meter (AquaLab 4TE, Meter Group, Inc., Pullman, WA, USA), which was previously used for the determination of pork‐related products. (Choudhury et al. 2019).

### 2.4 Puffing Ratio

Puffing ratio of pork rinds is determined by the difference in volume after and before frying using the sesame seed displacement technique (Chen et al. 2016).

### 2.5 Color Measurement

The International Commission on Illumination L*a*b* color space (CIELAB) of fried pork rind was collected by a spectrocolorimeter (TC‐1800 MK II, Tokyo denshoku, Tokyo, Japan) (Su et al. 2021), which is being calibrated with a white reference prior to the test.

### 2.6 Breaking Force Measure of Pork Rinds

The breaking force of fried pork rind was tested by the method of Su et al. (2016). A texture analyzer RapidTA+ (Horn Instruments Co., Ltd., Taoyuan City, Taiwan) was used to compress pork rinds at the pre‐, test‐, and post‐speed of 5.0 mm/s using a spherical stainless probe (P0.25 s). This apparatus was successfully used to determine the texture properties of pork rinds previously (Lin, Hou, and Sung 2021). The equipment underwent weight and height calibration using external weights and an internal calibration program before the test.

### 2.7 Scanning Electron Microscopy Observation

Fried pork rinds after lipid extraction (defatted) were cut to around 5 × 5 mm and stuck on brass stubs by a double‐sided carbon conductive tape. The samples were sputter‐coated (6 nm/min) with platinum using an ion sputter (Hitachi E‐1010, Hitachi Science System, Tokyo, Japan) for 60 s at 20 Pa and observed at 15.0 kV by a scanning electron microscope (SEM) (Hitachi S‐3400, Hitachi Science System, Tokyo, Japan) (Guo et al. 2024).

### 2.8 Statistical Analysis

The Box–Behnken method (Box and Behnken 2012) was conducted for the design of experiment using three independent elements, including drying time (X1: 12, 18, and 24 h), frying temperature (X2: 180, 195, and 210°C), and frying time (X3: 3, 4, and 5 min). RSM was performed using the Design Expert 12 software. One‐way analysis of variance was conducted by General Linear Model in the Statistical Products & Services Solution statistics software package version 25 (SPSS Inc., Chicago, Illinois, USA). Differences between averages were obtained by Ducan's multiple range test (*p* < 0.05). The analysis of physical properties of pork rinds was done in triplicate.

## 3 Results and Discussion

The results of nine response variables, including water and oil content; puffing ratio; breaking force; water activity; L*, a*, and b* values; and color difference of pork rinds under different processing conditions, are shown in Tables 1 and 2. The independent processing factors and physical properties agreed to the quadratic polynomial function and evaluated the coefficients of determination (*R* ^2^). The predicted regression models and ANOVA of all the variables reporting the attributes of drying time, frying temperature, and frying time on the quality characteristics of fried pork rinds are presented in Table 3. The *R* ^2^ values for water and oil content; puffing ratio; breaking force; water activity; L*, a*, and b* values; and color difference were 0.9111, 0.8416, 0.7576, 0.9312, 0.8954, 0.8191, 0.9656, 0.8960, and 0.7883, respectively. The better the predicted model fits the actual results, the closer the value of *R* ^2^ approaches unity. The predicted model for the quality property can be adopted to navigate the design space. Therefore, it can be adopted as a predictive model based on the coefficient of determination for the quality of fried pork rinds.

**TABLE 1:** Results of moisture content, oil content, puffing ratio, breaking force, and water activity of fried pork rinds on response surface methodology.

Run order	Drying time (h)	Frying temperature (°C)	Frying time (min)	Moisture content (g/100 g wb), *R*_1_	Oil content (g/100 g, db), *R*_2_	Puffing ratio (%), *R*_3_	Breaking force (N), *R*_4_	Water activity, *R*_5_
1	12	180	4	2.81 ± 0.84	24.15 ± 1.65	390.34 ± 27.95	65.495 ± 4.113	0.2393 ± 0.0232
2	18	180	3	2.55 ± 0.62	27.05 ± 1.86	517.47 ± 20.81	81.880 ± 7.061	0.2525 ± 0.0024
3	18	180	5	2.13 ± 0.06	35.40 ± 2.48	627.67 ± 26.93	49.071 ± 3.879	0.1907 ± 0.0037
4	24	180	4	1.73 ± 0.21	22.92 ± 3.24	601.37 ± 36.41	44.093 ± 7.691	0.2368 ± 0.0066
5	12	195	3	3.44 ± 0.10	32.46 ± 2.15	560.96 ± 33.13	58.907 ± 5.975	0.2468 ± 0.0085
6	12	195	5	1.43 ± 0.41	35.66 ± 2.32	496.20 ± 29.15	48.131 ± 8.983	0.2358 ± 0.0074
7	18	195	4	1.00 ± 0.21	35.55 ± 2.48	674.58 ± 35.76	34.004 ± 1.469	0.1969 ± 0.0044
8	18	195	4	1.69 ± 0.15	36.27 ± 1.92	702.43 ± 48.74	34.517 ± 4.038	0.2259 ± 0.0152
9	18	195	4	1.47 ± 0.19	35.88 ± 2.77	887.03 ± 75.33	35.064 ± 3.195	0.2030 ± 0.0190
10	24	195	3	1.86 ± 0.21	33.17 ± 2.51	948.09 ± 102.41	28.283 ± 4.801	0.2918 ± 0.0164
11	24	195	5	0.99 ± 0.15	40.98 ± 1.06	724.41 ± 71.93	31.628 ± 4.323	0.2366 ± 0.0094
12	12	210	4	1.22 ± 0.40	29.78 ± 8.61	541.75 ± 67.39	42.141 ± 5.347	0.1229 ± 0.0085
13	18	210	3	1.00 ± 0.23	25.52 ± 3.84	687.72 ± 93.95	38.841 ± 4.374	0.1973 ± 0.0107
14	18	210	5	0.66 ± 0.18	28.91 ± 1.39	754.45 ± 67.85	41.241 ± 2.476	0.1831 ± 0.0072
15	24	210	4	0.57 ± 0.25	29.97 ± 1.57	587.22 ± 54.24	39.442 ± 7.113	0.1634 ± 0.0076

*Note:* Expressed as mean ± standard deviation (*n* = 3).

**TABLE 2:** Results of L*, a*, b*, and color difference (Δ*E*) of fried pork rinds on response surface methodology.

Run order	Drying time (h)	Frying temperature (°C)	Frying time (min)	L*, *R*_6_	a*, *R*_7_	b*, *R*_8_	Color difference (Δ*E*), *R*_9_
1	12	180	4	69.00 ± 1.76	−8.50 ± 0.26	34.25 ± 0.81	20.84 ± 1.86
2	18	180	3	77.61 ± 3.49	−10.47 ± 0.44	35.15 ± 1.46	29.71 ± 3.66
3	18	180	5	73.19 ± 1.91	−9.55 ± 0.14	36.42 ± 0.50	25.49 ± 1.75
4	24	180	4	71.17 ± 2.19	−9.47 ± 0.21	35.22 ± 1.47	23.35 ± 1.81
5	12	195	3	76.55 ± 1.22	−8.73 ± 0.30	38.73 ± 1.30	29.11 ± 1.45
6	12	195	5	76.17 ± 1.30	−8.04 ± 0.53	42.62 ± 1.47	29.95 ± 1.54
7	18	195	4	76.94 ± 2.70	−9.22 ± 0.41	37.24 ± 0.91	29.32 ± 2.80
8	18	195	4	72.45 ± 1.13	−9.17 ± 0.15	35.53 ± 1.56	24.54 ± 1.32
9	18	195	4	75.34 ± 1.33	−9.94 ± 0.14	35.38 ± 0.57	27.43 ± 1.40
10	24	195	3	80.03 ± 1.45	−11.05 ± 0.45	37.92 ± 0.75	32.65 ± 1.37
11	24	195	5	70.65 ± 2.58	−10.16 ± 0.74	35.28 ± 0.45	22.98 ± 2.61
12	12	210	4	67.86 ± 3.14	−6.52 ± 0.12	42.89 ± 2.50	22.56 ± 3.97
13	18	210	3	75.53 ± 2.44	−8.80 ± 0.38	41.92 ± 0.62	29.19 ± 2.52
14	18	210	5	74.19 ± 2.29	−8.65 ± 0.75	40.68 ± 1.75	27.49 ± 2.79
15	24	210	4	74.58 ± 0.96	−8.83 ± 0.23	42.46 ± 1.22	28.55 ± 1.26

*Note:* Expressed as mean ± standard deviation (*n* = 3).

**TABLE 3:** Actual variable coefficients of physical properties of fried pork rinds.

	Intercept	*A*	*B*	*C*	*AB*	*AC*	*BC*	*A* ^2^	*B* ^2^	*C* ^2^
Responses	*r*_0_	*r*_a_	*r*_b_	*r*_c_	*r*_ab_	*r*_ac_	*r*_bc_	*r*_aa_	*r*_bb_	*r*_cc_
Moisture content	1.38667	−0.46875**	−0.72125**	−0.455**	0.1075	0.285	0.02	0.270417	−0.0745833	0.272917
Oil content	35.9	0.62375	0.5825	2.84375*	0.355	1.1525	−1.24	−1.42375	−7.77125**	1.09125
Puffing ratio	754.68	108.98*	54.2862	−13.9388	−41.39	−39.73	−10.8675	−94.4613	−130.049*	22.1962
Breaking force	34.5283	−8.9035**	−9.85925**	−4.73*	4.67575	3.53025	8.80225**	1.12171	12.1427**	6.08721
Water activity	0.2086	0.010475	−0.031575**	−0.017775*	0.01075	−0.01105	0.0119	0.014425	−0.032425**	0.029725*
L*	74.91	0.85625	0.14875	−1.94*	1.1375	−2.25	0.77	−1.76875	−2.48875	2.70875*
a*	−9.67667	−0.965**	0.64875**	0.33125**	−0.335	0.05	−0.1925	0.609583**	0.737083**	−0.427917*
b*	36.05	−0.95125	3.36375**	0.16	−0.35	−1.6325	−0.6275	1.375	1.28	1.2125
Color difference	27.0967	0.63	1.055	−1.845	0.8775	−2.6275	0.6325	−1.28708	−1.99208	2.86292*

*Note:* A: drying time (12–24 h); B: frying temperature (180–210°C); C: frying time (3–5 min). *R*_(i=1–9)_ = *r*_0_ + *r*_a_ *A* + *r*_b_ *B* + *r*_c_ *C* + *r*_ab_ *AB* + *r*_ac_ *AC* + *r*_bc_ *BC* + *r*_aa_ *A* ^2^ + *r*_bb_ *B* ^2^ + *r*_cc_ *C* ^2^, where r_0_ is an intercept constant; *r*_a_, *r*_b_, and *r*_c_ are linear coefficients; *r*_ab_, *r*_bc_, and *r*_ac_ are interaction coefficients; and *r*_aa_, *r*_bb_, and *r*_cc_ are quadratic coefficients.

* Indicate significance at 0.05 ≤ *p* < 0.1.

** Indicate significance at *p* < 0.05.

### 3.1 Water Content and Water Activity of Dried Pork Rinds

**FIGURE 1:**
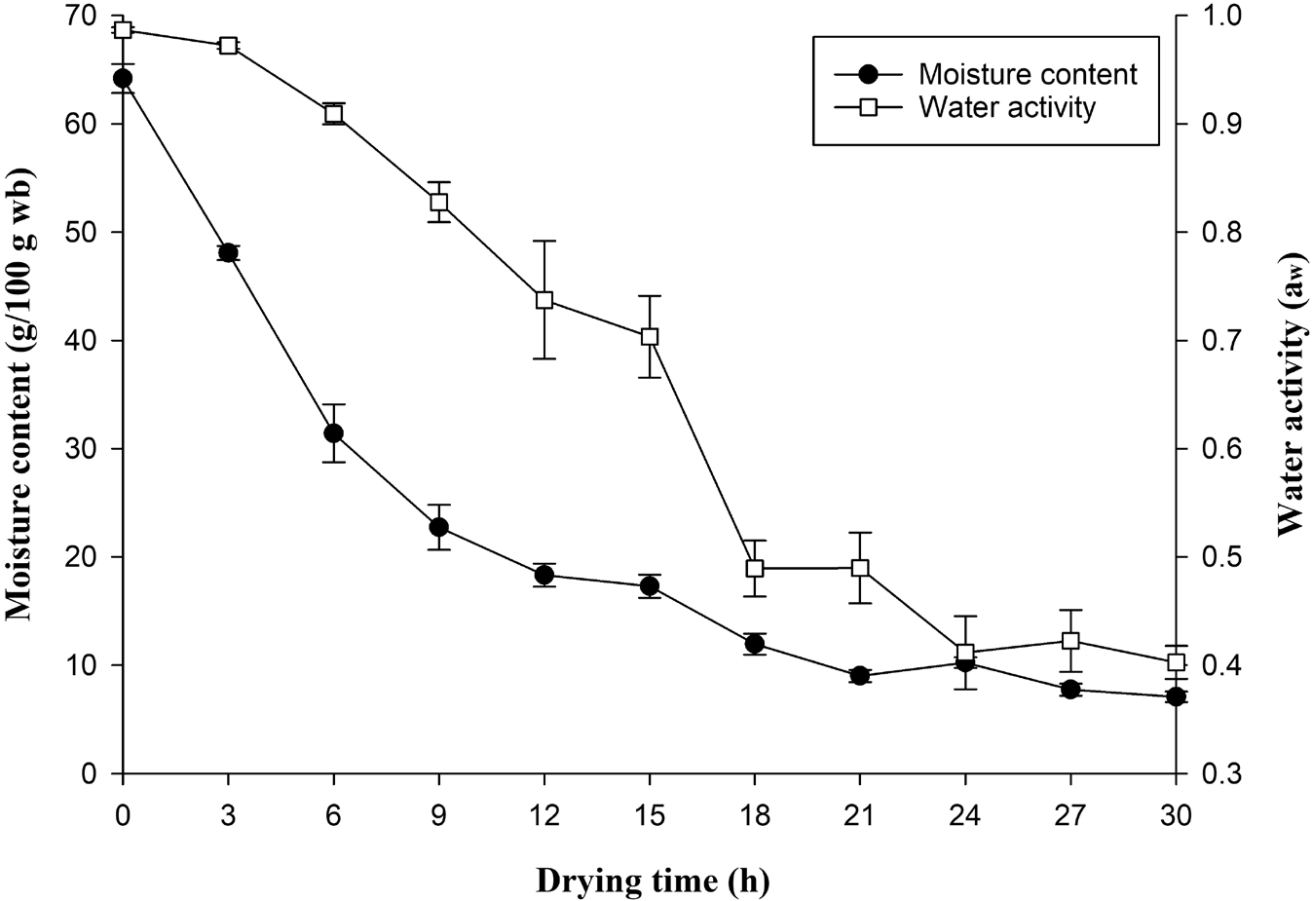
Changes on moisture content and water activity of pork skin by different drying times at 50°C.

The initial water content of the cooked pork rind was 64.19% (wet basis; % wb). It decreased during the first 9 h of hot‐air drying (22% wb, Figure 1), which is referred to the falling rate stage, and the trend was similar to the result of Krengkrat and Sriwattana (2020). The moisture content reached approximately 17.3% wb at the 15th h. The cooked pork rinds were mainly dehydrated at the falling rate stage. Heat evaporated the water of cooked pork rind and formed a case‐breaking force on the surface of dried pork rinds, with a faster level of moisture evaporation than that in the dehydrated pork rind at the final drying stage. Hot‐air drying at 50°C demonstrated slow dehydration during the final stage after 24 h and reached approximately 10.25% wb in 24 h. Taewee (2011) proposed that the moisture content of dehydrated rind reached the maximum frying pork rind puffing ratio in the range of 12%–15%. The water activity of the dried rind was below 0.6 after 18 h hot‐air drying at 50°C. Therefore, among the hot‐air drying times (12, 18, and 24 h with moisture of 18.33%, 11.96%, and 10.25% wb, respectively), 18 h was considered as the final drying time of the semiproduct processing.

The modeling of three processing factors, including drying time (A), frying temperature (B), and frying time (C), was performed via a three‐aspect, three‐degree Box–Behnken experimental design (Box and Behnken 2012) at the center point with three replicates using Design Expert 12.

The response function R_i_ is calculated follows:

$$\begin{array}{c} R_{\left(i=1–9\right)}=r_{0}+r_{a}A+r_{b}B+r_{c}C+r_{\mathrm{ab}}\mathrm{AB}+r_{\mathrm{ac}}\mathrm{AC}+r_{\mathrm{bc}}\mathrm{BC}+r_{\mathrm{aa}}A^{2}\\ \;+r_{\mathrm{bb}}B^{2}+r_{\mathrm{cc}}C^{2} \end{array}$$

where the response function *R* (*i* = 1–9) is the estimated response for water content; oil content; puffing ratio; breaking force; water activity; L*, a*, and b* values; and color difference. *R* ^2^ describes the predicted regression coefficient of the adequate response in the Box–Behnken design (BBD); *r*_a_, *r*_b_, and *r*_c_ depict the regression coefficient for linear effect items. *A* depicts the drying time; *B* depicts the frying temperature; and *C* depicts the frying time. *r*_0_ is the intercept constant; *r*_ab_, *r*_bc_, and *r*_ac_ are the interaction coefficients; and *r*_aa_, *r*_bb_, and *r*_cc_ are the quadratic coefficients. The responses tested included water content; oil content; puffing ratio; breaking force; water activity; L*, a*, and b* values; and color difference. The significance of levels in the polynomial function was determined by calculating the probability (*p*) of 0.1 and 0.05. Then, the regression coefficients were adopted to perform statistical analysis and to draw the response surface plot.

Tables 1 and 2 demonstrates the averages of the physical qualities of fried pork rinds. The estimated ANOVA and regression models of the terms explaining the effect of drying time and frying factors on the water content of the pork rinds are presented as follows:

$$\begin{array}{c} R_{1}=1.39-0.4688A-0.7213B-0.4550C+0.1075\mathrm{AB}+0.2850\mathrm{AC}\\ \;+0.0200\mathrm{BC}+0.2704A^{2}-0.0746B^{2}+0.2729C^{2} \end{array}$$

The coefficients of determination (*R* ^2^) and adjusted *R* ^2^ were 0.9111 and 0.7510, respectively. The developed model can be adopted to navigate the design space; thus, it can be adopted as a predictive model for the moisture content of pork rinds. Furthermore, the three variables (drying time, frying temperature, and frying time) presented a significant fit (*p* < 0.05); hence, the model is a suitable index of the responses, and it can be adopted for estimation (Myers, Montgomery, and Anderson‐cook 2009). A second‐order polynomial equation of independent factors suitable for each response analyzed the oil content of fried pork rind, which is presented as follows:

$$\begin{array}{c} R_{2}=35.90+0.6237A+0.5825B+2.84C+0.3550\mathrm{AB}+1.15\mathrm{AC}\\ \;-1.24\mathrm{BC}-1.42A^{2}-7.77B^{2}+1.09C^{2} \end{array}$$

**FIGURE 2:**
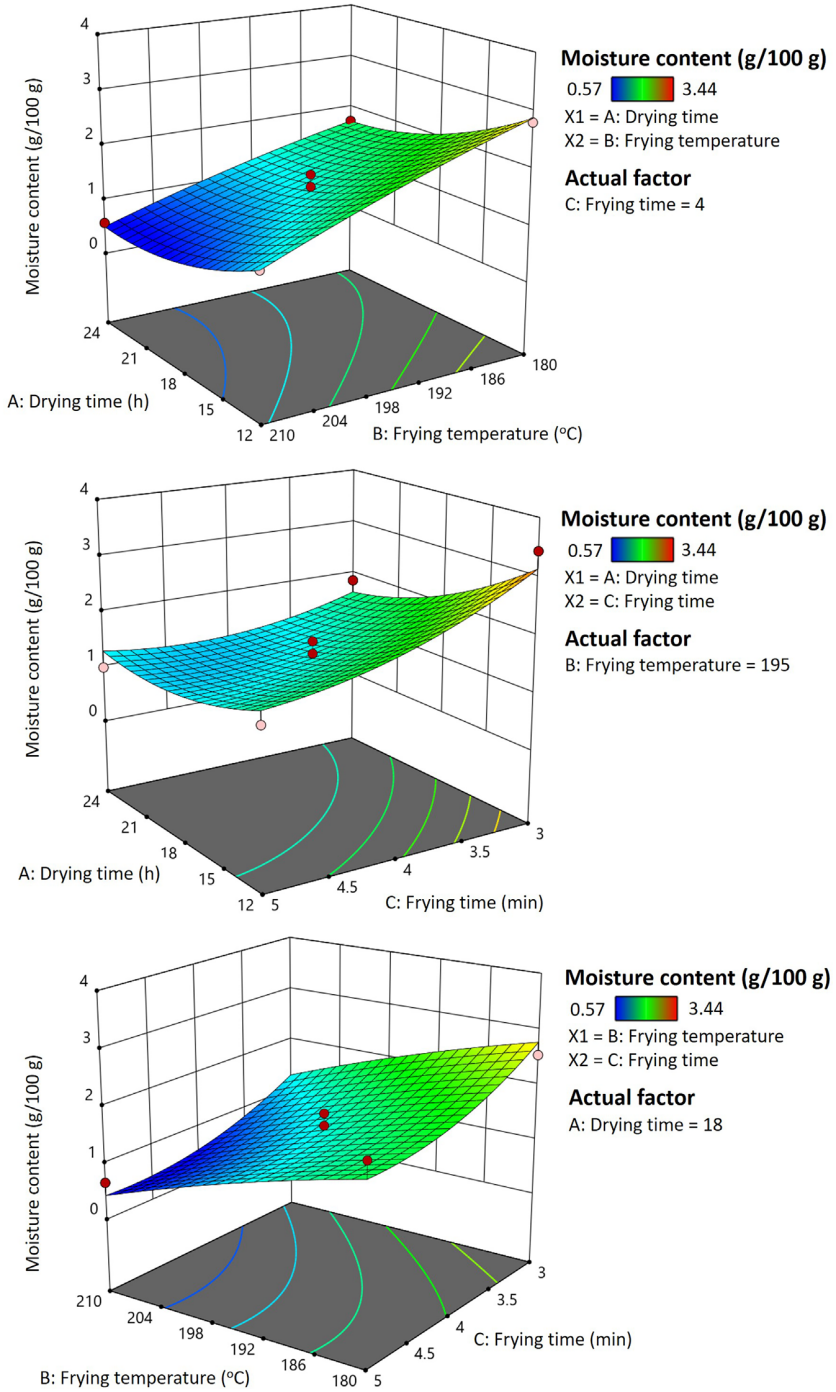
Response surface plot for the effect of different drying times, frying temperatures, and frying times on the moisture content of fried pork rinds.

At the same frying temperature and time, the water content of dried pork rind reduced by increasing the drying time, which developed in the low water content of pork rinds. The contour plot and response surface plot show that the moisture content of pork rinds reduces with the elongation of drying time, frying temperature, and time (Figure 2). This finding was comparable with the data observed by Southern et al. (2000), who proposed that higher frying temperatures and longer frying times increase the evaporation of the moisture content of fried pork rind and heat transfer of deep frying. The reported drying time for cooked pork rind through higher frying temperature and longer frying time for deep frying obtained low moisture‐fried pork rinds.

The water activity of dried pork rind subjected to traditional deep frying decreased faster in the first 3 min than hot‐air drying because of the rapid thermal transfer by oil than hot air. Hot oil could also get in the interstices in the pork rind by capillary action. Low‐temperature drying time did not significantly affect the water activity of pork rinds (*p* > 0.05; data not shown). It took 24 h to achieve a water activity level of 0.4, which is a relatively slow process from a food safety perspective compared to deep frying (Figure 1). Deep frying, on the other hand, showed a rapid decrease in water activity within the first 3.5 min, after which the rate of decrease resembled that of traditional deep frying at 180°C (Lin, Hou, and Sung 2021). The lower water activity of the pork rinds obtained was primarily attributed to the higher frying temperature, rather than to frying time or drying time (Appendix S1).

### 3.2 Oil Absorption

**FIGURE 3:**
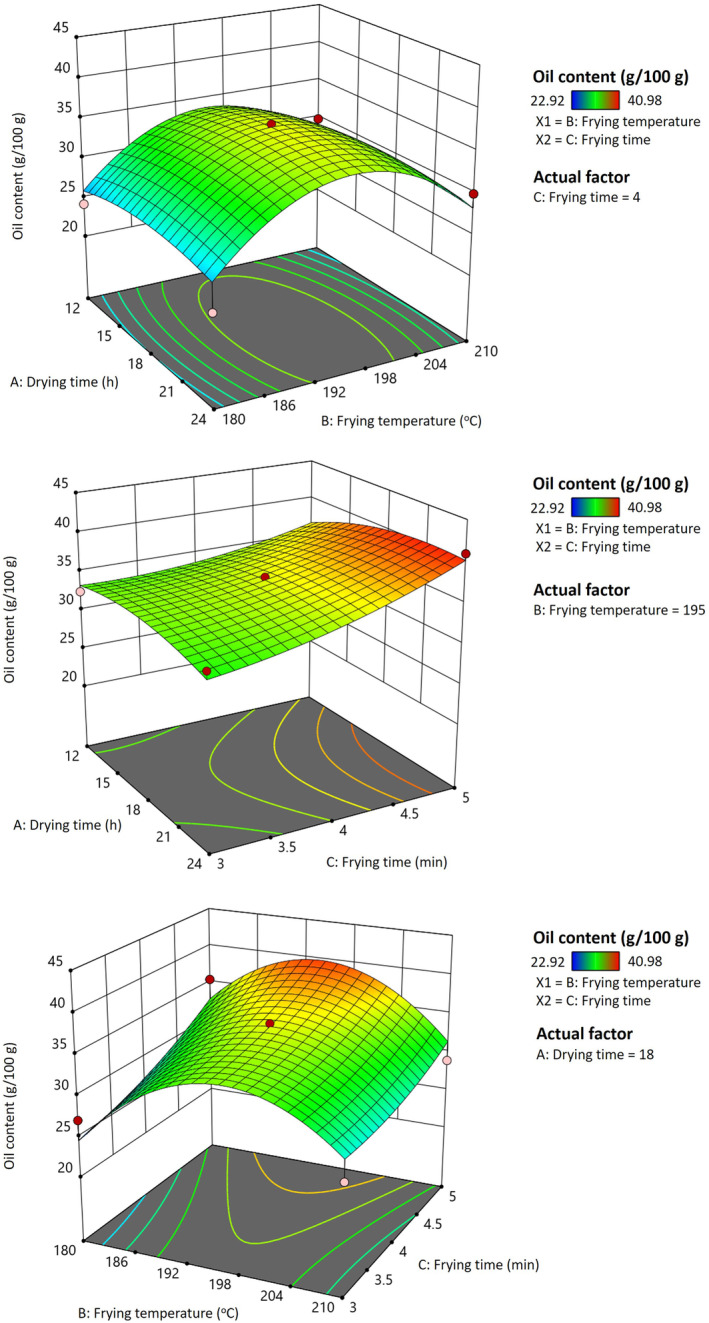
Response surface plot for the effect of different drying times, frying temperatures, and frying times on the oil content of fried pork rinds.

Figure 3 demonstrates a three‐dimensional plot illustrating the oil content of fried pork rinds as influenced by drying time, frying time, and temperature. The oil content of pork rinds was positively and linearly related to the frying time (0.05 < *p* < 0.1; *p* = 0.0681 data not shown). However, drying time had no significant effect on the oil content of the pork rinds (Figure 3), consistent with the findings from Liu et al. (2021). This lack of influence may be due to the minimal differences in water content among the dried pork rinds. The oil content of fried pork rinds increased with the increase of frying temperature up to 195°C but then decreased as both the frying time and temperature continued to rise. This could be explained by the high puffing ratio, which creates more holes and cracks on the surface of the rinds, allowing more frying oil to be absorbed as they cool down after frying (Isik, Sahin, and Sumnu 2016). Pork rinds that were hot‐air‐dried for 18 h and fried at 180°C had a higher oil content than those fried at 210°C (Figure 3). This result may be because a higher frying temperature causes case hardening on the surface of pork rind, which mitigates oil absorption during frying and cooling down. A similar effect of oil content was observed by Dehghannya, Naghavi, and Ghanbarzadeh (2016) in potato strips. However, other studies (Ziaiifar, Courtois, and Trystram 2010; Bouchon, Aguilera, and Pyle 2003) have reported that increasing frying temperatures also led to higher oil content in fried food, although these changes were not statistically significant, contrasting with our results.

The oil content of dried pork rind was approximately 5.19% wb (Lin, Hou, and Sung 2021). The oil uptake of pork rinds in the traditional deep‐fat frying method is primarily due to the pressure difference between the pork rind surface and inside when the samples leave the frying oil and cool down. The oil is taken up into the food interior from the cracks and holes in the food (Ufheil and Escher 1996). A similar result of oil uptake was reported in starchy food, and the oil absorption can be mitigated by drying before frying (Troncoso and Pedreschi 2009). Nevertheless, the oil content of pork rinds was not significantly influenced by drying time based on our results (Figure 3). A similar result of oil absorption was reported in potato strips (Liu et al. 2021), which is consistent with our data.

### 3.3 Changes in Breaking Force, Puffing Ratio, and Color of Fried Pork Sink During Frying

**FIGURE 4:**
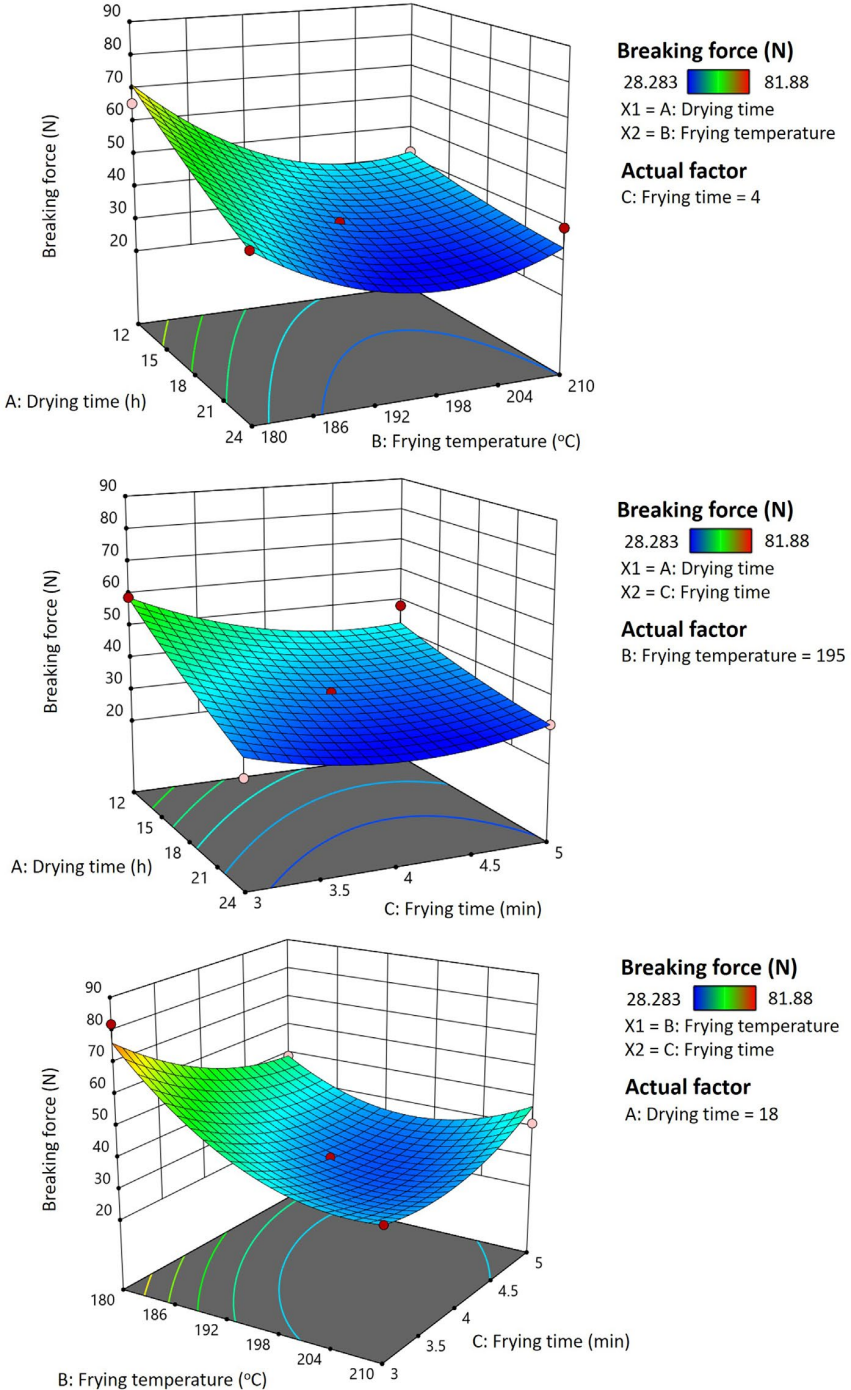
Response surface plot for the effect of different drying times, frying temperatures, and frying times on the breaking force of fried pork rinds.

Hardness is related to the breaking force and crispiness of the fried product (Ngadi, Adedeji, and Kassama 2009). Figure 4 demonstrates that drying time and frying temperature played critical roles in the breaking force (*p* < 0.05). The breaking force decreased as drying time increased; however, it initially decreased and then increased with rising frying temperatures. These findings were opposite to the trends observed for the puffing ratio. Thus, enhancing the puffing ratio of pork rinds leads to a reduction in their breaking force. (Van Koerten et al. 2015). When the frying temperature was under 195°C, the breaking force reduced as the frying time increased. When the frying temperature is more than 195°C, the breaking force increases with the frying time. High frying temperatures induce protein denaturation and dehydration, making the texture firm, and case hardening hinders the rind puff and increases the breaking force of pork rinds during further frying as the water content decreased to about 3%. The increase in the breaking force was because of the drying‐related denaturation of collagen and protein. The subsequent increase in the breaking force was due to the drying‐related denaturation of protein and collagen in the pork rind. An interaction was found between the breaking force of pork rinds as a function of frying temperature and frying time (*p* < 0.05; data not shown). The quadratic effect of the frying temperature was shown with the breaking force of the pork rinds (*p* < 0.05).

The breaking force and appearance of pork rinds are important and are characterized by the formation of a crispy texture, protein denaturalization, expansion, water evaporation, oil ingress, and tissue browning, which are the most appreciated factors by consumers (Troung et al., 2014).

**FIGURE 5:**
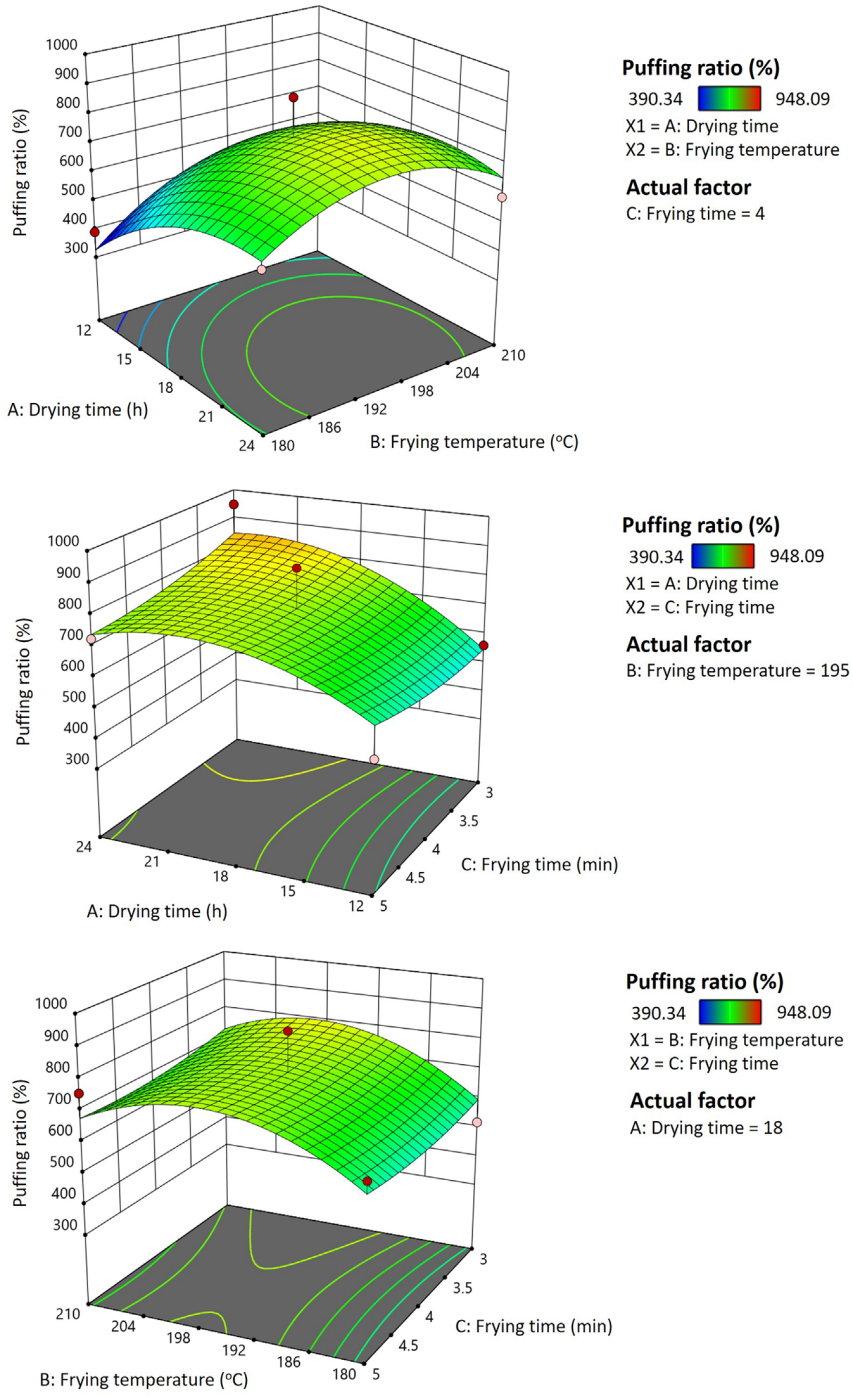
Response surface plot for the effect of different drying times, frying temperatures, and frying times on the puffing ratio of fried pork rinds.

Response surface model parameters indicated that the puffing ratio of pork rinds was linearly related to drying time, with a significance level of 0.05 < *p* < 0.1. Based on the obtained results, the best puffing ratio (> 700%) of the optimized pork rind conditions is obtained at drying times in the range of 15–24 h at 50°C and frying temperatures in the range of 190°C–205°C (Figure 5). The processing conditions that resulted in the best puffing ratio are similar to the results of Troung et al. (2014) at 14% water content of the dried pork rind and 190°C frying temperature. During hot‐air drying, a dehydrated and denatured crust was generated on the surface of the rinds, and the moisture inside the pork rind migrated slowly to the surface of the pork rind. The frying oil heated up the remaining moisture into steam and puffed up the rind's structure until the water content reduced to ca. 3%. During deep frying, the steam shifted into the interior of the pieces of the rind, generating a crust behind. As frying continued, the moisture content further decreased, thereby causing the evaporation of steam to stop. As shown in Figure 5a, the puffing ratio increases with drying time compared with various frying temperatures. During deep frying, the moisture of the dried pork rind is heated in a closed environment where force convection heat transfer is introduced. When the water content is too high or too low, the puffing ratio decreases. The high moisture content in dehydrated pork rind will generate insufficient steam pressure to blow up the compact interior pork rind structure. It could be improved by increasing the frying temperature to increase the steam‐generating rate. Nevertheless, an excessively high oil temperature (> 200°C) will speed up the case‐hardening structure and slow down steam release, causing protein denaturation and decreasing the puffing ratio of the fried pork rind. The puffing ratio of pork rinds decreased as the frying time increased (Figure 5b). Lin, Hou, and Sung (2021) reported that the puffing ratio of fried pork rinds reached its maximum after 1 min. Ziaiifar, Courtois, and Trystram (2010) proposed that the fried pork rind structure will partially collapse and form more holes during cooling. The low moisture content in dried pork rind will not have enough steam to puff the structure of pork rinds (Lee et al. 2000). The puffing ratio and pore structure of pork rinds decreased with extended frying time, likely due to a reduction in pore volume. This decline is attributed to prolonged frying, which leads to the deterioration of the collagenous connecting tissue, resulting in protein shrinkage, denaturation, gelation, and agglomeration, as well as increased oil absorption and moisture loss during deep frying.

The results of ANOVA and regression analysis for the color parameters of the response surface model on fried pork rinds showed that the frying time had a significantly linear effect on the L* value and color difference of fried pork rinds (0.05 < *p* < 0.1). The drying time had a strong linear relationship with the a* value of fried pork rind samples (*p* < 0.05), followed by the interaction factors of frying time and temperature (0.05 < *p* < 0.1). Frying temperature had a strong linear effect on the b* value of fried pork rinds (*p* < 0.05). Fried pork rind demonstrated higher L* and lower a* values than dried pork rind (data not shown). The L* value decreased with shorter drying time, lower frying temperature, and increasing frying time. The color difference decreased with the increase of frying time, and it was more evident when the drying time increased and the frying temperature decreased. The color difference change trend is similar to the L* value change; therefore, we hypothesized that the L* value has a great effect on the color difference. This result was due to the puffing effect of pork rind resulting from the L* value of the lightened pork rind. The decrease in a* value was due to the effect of two forms of melanins, namely, pheomelanins (yellow/red pigment) and eumelanins (black/brown pigments), in pork rind modified during 1 h of cooking and deep‐fat frying, which lightened and turned the pork rind greenish (Fontanesi and Russo 2013). Color is considered as a critical property of fried pork rinds, which is influenced by the frying oil type, quality, frying temperature, frying time, pretreatment conditions, and food composition (Yang et al. 2021). It is also influenced by other factors such as browning reaction during frying, oil uptake, and density changes of fried products (Altunakar, Sahin, and Sumnu 2004). Kitpot et al. (2019) revealed that the brighter appearance of fried pork rinds is correlated to the higher overall acceptance by the consumers. Therefore, deep‐fat frying at a higher frying temperature increased the nonenzymatic browning appearance of fried pork rind, resulting in reddish and yellowish colors on the surface, which should be avoided during the frying process.

Pork rind puffing ratio was not affected by the drying time of pork rind at a high frying temperature (210°C), and its color turned yellow. When the pork rind was fried at a low frying temperature (180°C), the increasing drying time decreased the water content of the dried pork rind and increased the puffing ratio of pork rinds, which decreased the dark brown portion of the unpuffed pork rind and lightened the color of the fried pork rind. Additionally, the analysis of variance and regression for response surface model parameters affecting each quality index of fried pork rinds are provided as Appendix S2a‐i.

### 3.4 Microstructure and Overall Properties of the Fried Pork Rinds

**FIGURE 6:**
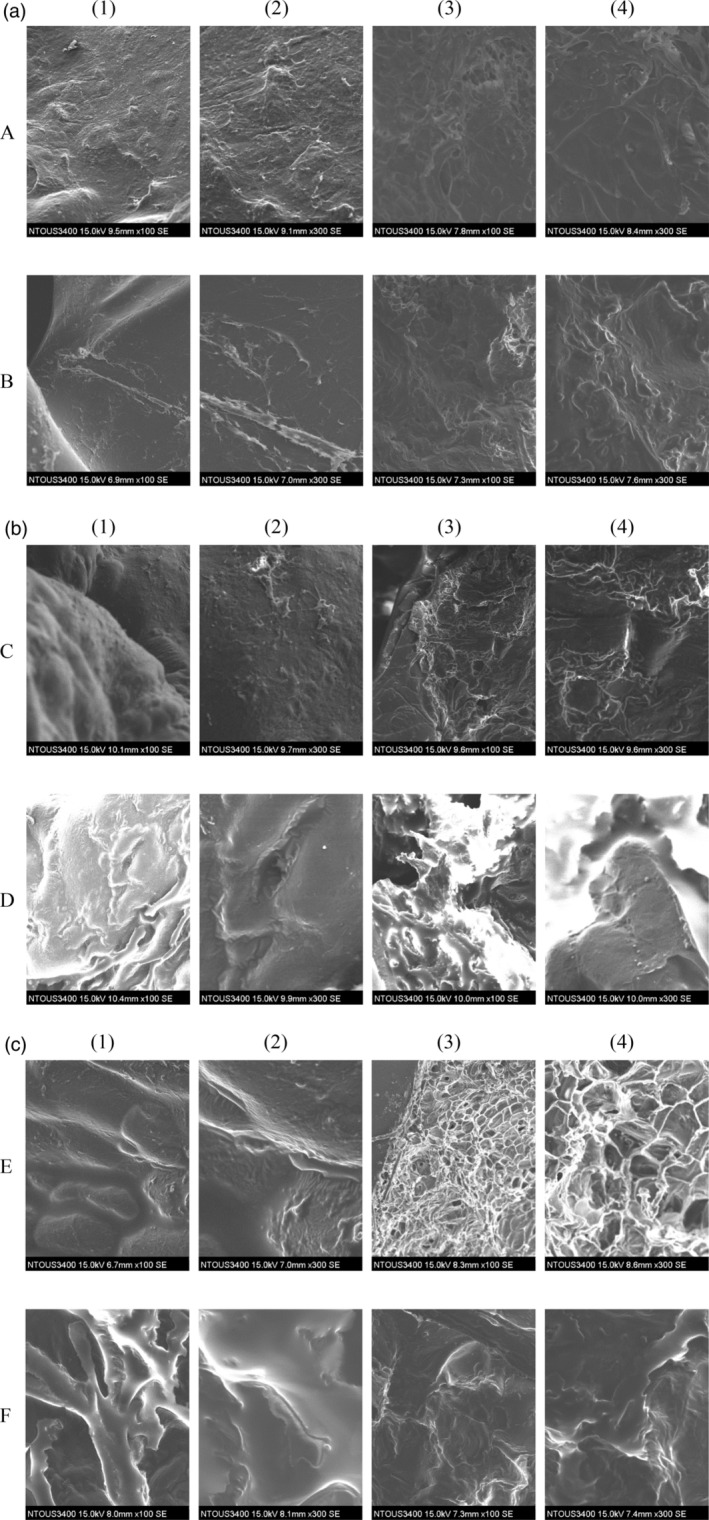
Scanning electron microscopy photograph of fried pork rinds by different processing conditions. The magnification of images was set as 100 and 300. (a)—(A) Drying time 12 h, frying temperature 180°C, and frying time 4 min; (B) Drying time 24 h, frying temperature 180°C, and frying time 4 min. Epidermis layer: (1) ×100 and (2) ×300; subcutaneous layer: (3) ×100 and (4) ×300. (b)—(C) Drying time 24 h, frying temperature 195°C, and frying time 5 min; (D) Drying time 22.35 h, frying temperature 197°C, and frying time 3.8 min. Epidermis layer: (1) ×100 and (2) ×300; subcutaneous layer: (3) ×100 and (4) ×300. (c)—(E) Drying time 24 h, frying temperature 210°C, and frying time 3 min; (F) Drying time 18 h, frying temperature 210°C, and frying time 5 min. Epidermis layer: (1) ×100 and (2) ×300; subcutaneous layer: (3) ×100 and (4) ×300.

**FIGURE 7:**
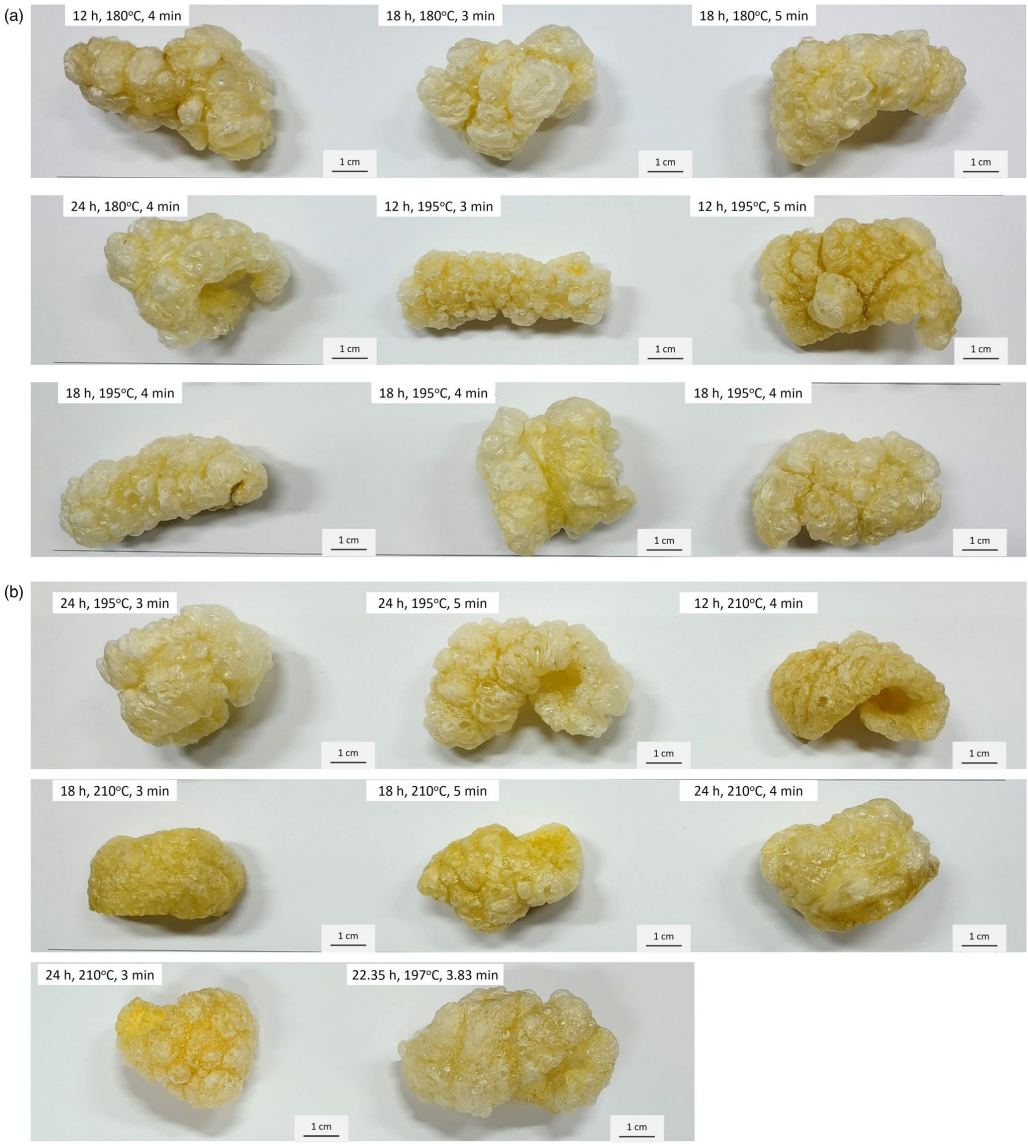
(a, b) Appearance of fried pork rinds by different processing conditions.

The ideal frying times were based on safety issues (water activity of < 0.3) and physical properties. Nonuniform holey and irregular structures were observed under the scanning electron micrographs of fried pork rinds prepared by traditional deep frying (Figure 6). The holes may have been generated by spontaneous water vapor formation, protein dehydration, denaturation, and uptake of oil during deep frying at different frying temperatures. Water absorbed energy from frying oil, and they were activated during deep frying resulting in spontaneous transfer through the protein structure, partially inducing their collapse and forming nonuniform holes. Pork rind fried at 210°C (Figure 7) demonstrated integrity and a regular shape. This result is due to the fast transfer of mass and heat at high oil temperatures. The heat flux from hot oil rapidly transferred through the dried or partially denatured solid crust of pork rind to the interior of the rind. The overheated surface and formed dryer outside had a fast water removal pace in deep‐fat fried pork rinds. Faster moisture and heat transfer reduces the collapse of the cellular structure and maintains the integrity of the cell, especially on the surface.

Figure 7 shows that the surface of pork rinds fried at a higher temperature (210°C) contains several small and more uniform bubble structures, which denature and maintain their structure quickly, and these air cells can maintain their frame after cooling compared with low‐temperature frying (180°C). Frying at a low temperature, the moisture evaporates slowly, and a large hollow empty bubble structure is formed, which collapses and shrinks after cooling, thereby decreasing the puffing ratio.

Figure 6 (1) and (2) columns illustrate that the epidermis layer of the fried pork rinds exhibits a uniform distribution of air cells and a smooth, porous structure. In contrast, Figure 6(3) and (4) column show that the subcutaneous layer is less uniform, featuring numerous rough holes and cracks on the exterior, as depicted in Figure 7. The epidermis layer of the fried pork rind was denser, and it showed less cracks compared with the subcutaneous layer. The smooth epidermis layer surface rolls up into the fried pork rinds (Figure 7) to mitigate the absorption of oil during frying and cooling (Liu et al. 2021). Therefore, we proposed that oil absorption occurs mainly at the subcutaneous layer. Fewer holes and cracks were also found when the pork rind was fried at 180°C and 210°C Figure 6(a) and Figure 6(c) than when the pork rind was fried at 195°C (Figure 6b). Thus, pork rind fried at 195°C contains more oil. In addition, at a similar puffing ratio of fried pork rinds containing more oil content, the surface and interior of pork rinds have more irregular large air cells and cracks.

### 3.5 Optimization and Comparison Between the Predicted and Experimental Results of Pork Rinds

In pork rind frying, the pork rind must be cooked, dried, and fried under optimum conditions to obtain desirable fried pork rinds. Therefore, by considering the importance of obtaining the optimal processing conditions for frying pork rind via hot‐air drying and deep frying conditions, the best combination of process factors (drying time, frying temperature, and frying time) is evaluated. The optimal processing conditions were predicted and compared with the experimental results by superimposing all the contour plots of these three factors. The first optimum processing condition and breaking force optimal conditions would be considered if water, fat content, and breaking force of pork rinds were low and if their puffing ratio was high. The second optimum process condition would be the commercial fried pork rind quality if the fat content was set to 35 g/100 g db, the puffing ratio was set to maximum, and its breaking force was set to minimum. Two sets of predicted and desirability values of optimal conditions are shown in Appendices S3 and S4. The two predicted and experimental results of the optimal conditions for the RSM of fried pork rinds are shown in Appendix S5. Due to the desirability methodology, two optimal processing condition settings of the experimental elements involving water and oil content, breaking force, and puffing ratio of fried pork rinds were obtained at the drying time of 24 h, frying temperature of 210°C, and frying time of 3.8 min. At these settings, the experimental results fell within the 95% prediction interval (Appendix S5) compared with the predicted and commercial pork rinds. Although the experimental puffing ratio and breaking force of pork rinds in the optimal processing condition 1 were low, the BBD had poor prediction at extreme points (Yolmeh and Jafari 2017). The experimental oil content (40.37%) of pork rinds was higher than that (34.91%) of commercial fried pork rinds (Appendix S5). Therefore, there are other factors influencing the oil content of fried pork rinds, which are not considered in the experiment. However, the optimal conditions for the RSM of fried pork rinds had a well‐fitted model near the predicted qualities, whereas the desired maximum puffing ratio and minimum moisture and oil contents were at very low and high extreme points. The predicted results for the experimental design still need some further adjustments.

As shown in Table 3, the frying temperature has a curvature trend on the oil content (*p* < 0.05), puffing ratio (0.05 < *p* < 0.1), and breaking force (*p* < 0.05) of fried pork rinds. Drying time has a positively linear effect on the puffing ratio (−0.05 < *p* < 0.1) of fried pork rind samples (Table 3). Frying time has a positively linear effect on the oil content (0.05 < *p* < 0.1) and a negatively linear effect on the breaking force (0.05 < *p* < 0.1) of pork rinds (Table 3). Therefore, we can first select a frying temperature near or away from our target physical properties and then select drying and frying times to approach our target qualities. Table 4 presents seven predicted results of optimal conditions for the RSM of fried pork rinds. Optimal condition 3 predicted the minimal oil content; optimal condition 4 predicted the maximal puffing ratio; and optimal condition 5 predicted the minimal breaking force for fried pork rinds (Table 4). In addition, frying at 195°C can decrease the breaking force and increase the puffing ratio but increase the oil content of the fried pork rind. Nevertheless, increased frying time led to decreased breaking force and increased oil content of the pork rinds. To reduce the processing time, such as by shortening the drying time (as in optimal conditions 6 and 7), the frying temperature (in optimal condition 7) or frying time (in optimal condition 6) should be increased to enhance the puffing ratio and decrease the breaking force of the fried pork rinds (Table 4). Sriwattana et al. (2012) employed a different approach, directly linking moisture content, fat content, and hardness to consumer acceptability. Their findings indicated that fried pork rinds with lower moisture, fat content, and hardness received higher overall liking. In line with these insights, our model suggests that lower frying temperatures (< 180°C) combined with extended drying times (> 18 h) are optimal. These conditions produce pork rinds with low moisture content, reduced fat, minimal breaking force, and a higher puffing ratio. However, considering the variations in fat content preferences across different countries and regions, we referenced the fat content of commercially available fried pork skin products (35%) as a benchmark (Table 5). As a result, the predicted optimal conditions should be broadly appealing to consumers.

**TABLE 4:** Predicted results of optimal conditions for the response surface methodology of fried pork rinds.

Goal	Processing conditions (drying time, frying temperature, and frying time)	Oil content (dry basis, g/100 g)	Puffing ratio (%)	Breaking force (*N*)
Optimal condition 1	24 h, 210°C, 3 min	26.60	738.78	32.191
Optimal condition 2	22.35 h, 197°C, 3.8 min	34.91	794.69	28.459
Optimal condition 3	23.60 h, 180°C, 3 min	22.49	690.75	61.158
Optimal condition 4	22.55 h, 197°C, 3 min	33.07	851.46	34.762
Optimal condition 5	23.20 h, 196°C, 4.1 min	35.40	778.83	27.320
Optimal condition 6	14.88 h, 199°C, 4.9 min	37.64	704.50	38.095
Optimal condition 7	15.71 h, 210°C, 3.3 min	27.85	656.99	39.664

*Note:* Optimal condition 1: minimize oil content, maximize puffing ratio, and minimize breaking force. Optimal condition 2: oil content = 35, maximize puffing ratio, and minimize breaking force. Optimal condition 3: minimize oil content. Optimal condition 4: maximize puffing ratio. Optimal condition 5: minimize breaking force. Optimal condition 6: minimize drying time, maximize puffing, and minimize breaking. Optimal condition 7: minimize drying time, minimize oil, maximize puffing, and minimize breaking force.

**TABLE 5:** Scores of the sensory evaluation of fried pork rinds under different processing conditions.

Drying time (h)	Frying temperature (°C)	Frying time (min)	Appearance	Odor	Flavor	Texture	Greasy intensity	Overall acceptability
Commercial			6.24 ± 2.20^a^	5.38 ± 2.32^a^	5.88 ± 2.17^a^	6.40 ± 2.15^a^	3.96 ± 2.29^a^	5.96 ± 2.02^a^
12	180	3	5.53 ± 1.74^b^	5.46 ± 1.91^ab^	5.78 ± 1.93^a^	5.31 ± 2.22^b^	4.14 ± 2.26^a^	5.29 ± 2.18^b^
22.35	197	3.8	6.11 ± 1.39^a^	6.13 ± 1.72^b^	6.21 ± 2.03^a^	6.78 ± 1.71^a^	3.64 ± 2.11^a^	6.32 ± 1.91^a^
24	210	3	6.18 ± 1.68^a^	5.90 ± 2.02^ab^	6.14 ± 1.91^a^	6.90 ± 1.65^a^	3.83 ± 2.20^a^	6.21 ± 1.92^a^

*Note:* Expressed as mean ± standard deviation (*n* = 72). Values followed by different letters within each column are significantly different (*p* < 0.05). Using a 9‐point scale hedonic test for appearance, odor, flavor, texture, and overall acceptability: 1 = dislike extremely, 5 = neither like nor dislike, and 9 = like extremely. Using a 9‐point scale hedonic test for greasy intensity: 1 = no greasy and 9 = strong greasy.

## 4 Conclusions

This research aimed to evaluate the quality of cooked pork rind samples by analyzing their oil content, moisture content, breaking force, color, puffing ratio, and microstructural appearance. The samples were subjected to various drying times (12–24 h at 50°C) and fried at different temperatures (180°C–210°C) and durations (3–5 min), with the analysis conducted using RSM. Frying temperature significantly influenced the oil content, puffing ratio, and breaking force of the fried pork rinds, with these variables fitting a second‐order polynomial equation by multiple regression analysis. The effect of dehydration time on the puffing ratio and breaking force fitted a linear model. Increasing drying time and frying temperature increased the puffing ratio and decreased the breaking force of the fried pork rinds. However, frying at 195°C resulted in a higher oil content. The obtained optimum process conditions indicate that the results fell within the 95% prediction interval. Response surface optimization is a viable method for preparing fried pork rinds with specific desired characteristics for the food industry.

## Author Contributions

**Shang‐Ta Wang:** conceptualization (equal), data curation (equal), writing – review and editing (equal). **Hong‐Ting Victor Lin:** formal analysis (equal), writing – original draft (equal). **Yu‐Jin Syu:** data curation (equal), formal analysis (equal), investigation (equal). **Wen‐Chieh Sung:** conceptualization, writing‐review and editing, project administration, resources, supervision.

## Ethics Statement

The authors have nothing to report.

## Consent

Written informed consent was obtained from all study participants.

## Conflicts of Interest

The authors declare no conflicts of interest.

## Supporting information


Appendix S1.

## Data Availability

The authors have nothing to report.
