# Supplementary material for: Effect of Drying Time and Frying Conditions on the Quality of Pork Rinds by Response Surface Methodology
Source: Food Sci Nutr. 2024 Nov 1;12(12):10194–210. doi: 10.1002/fsn3.4513 (PMC11666996; doi:10.1002/fsn3.4513)
Supplement: Supplementary file 1 — Appendix S1. [file FSN3-12-10194-s001.docx]

| 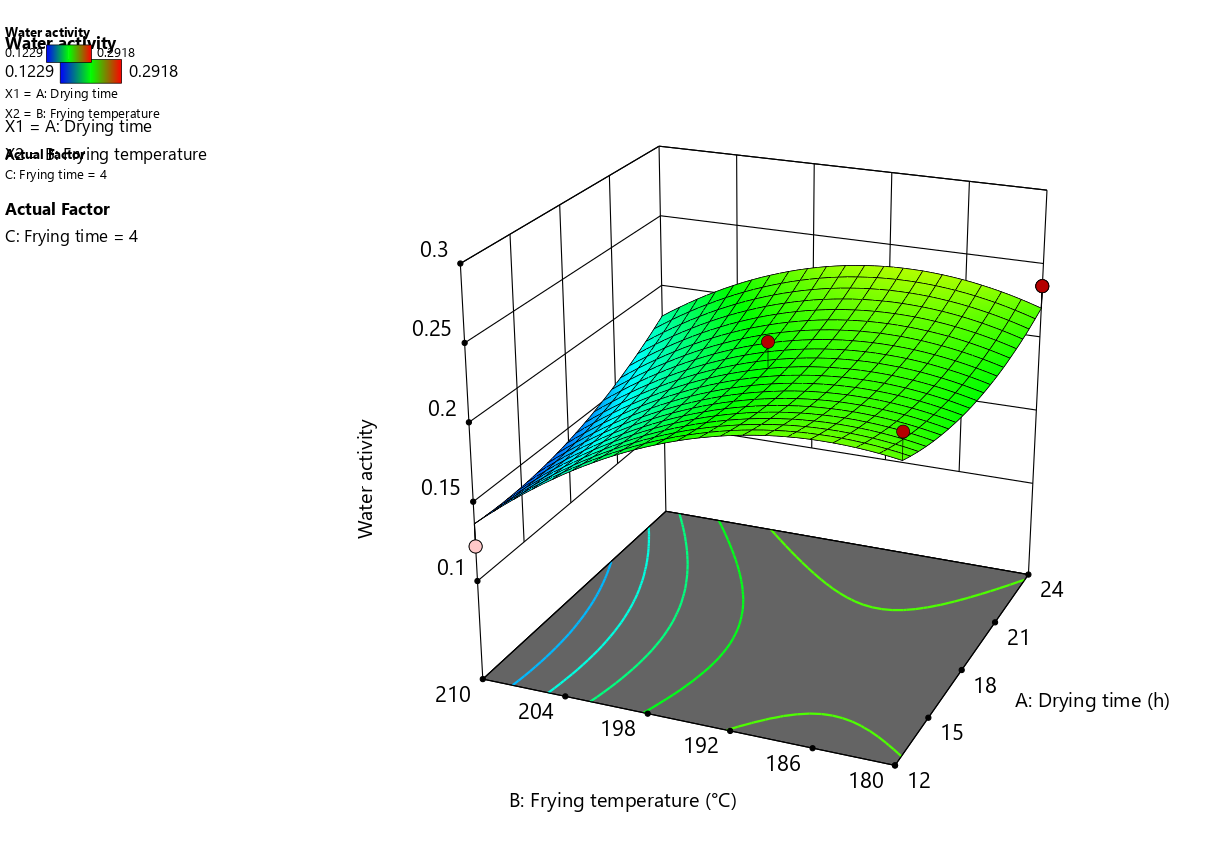 | 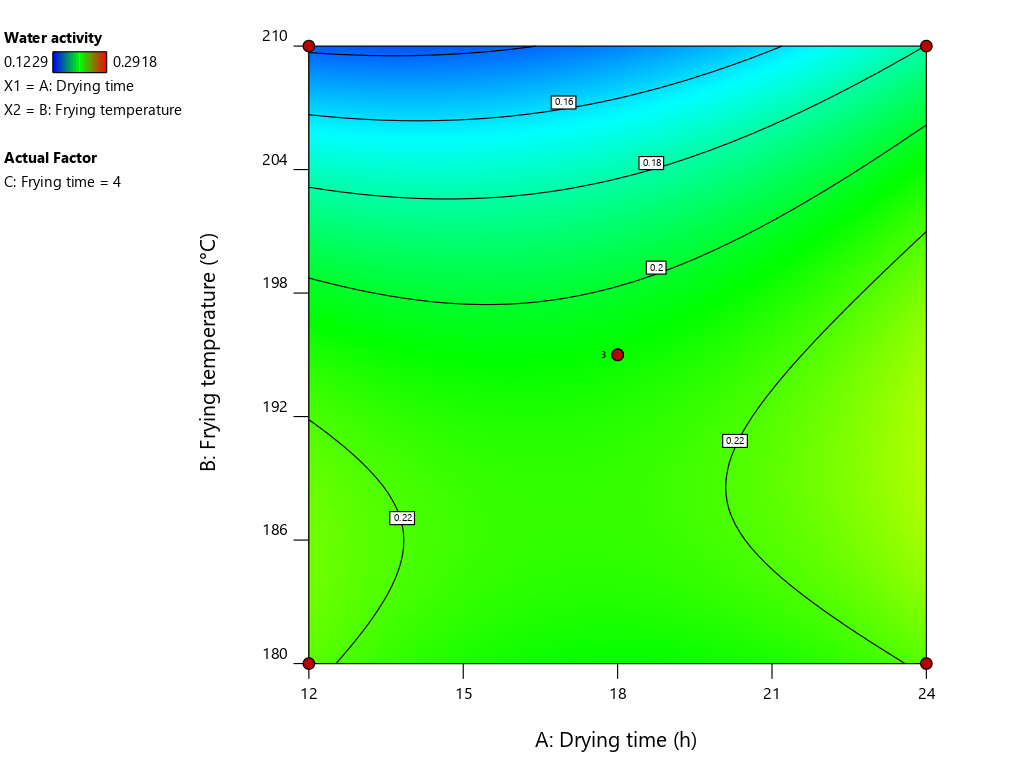 |
| --- | --- |
| 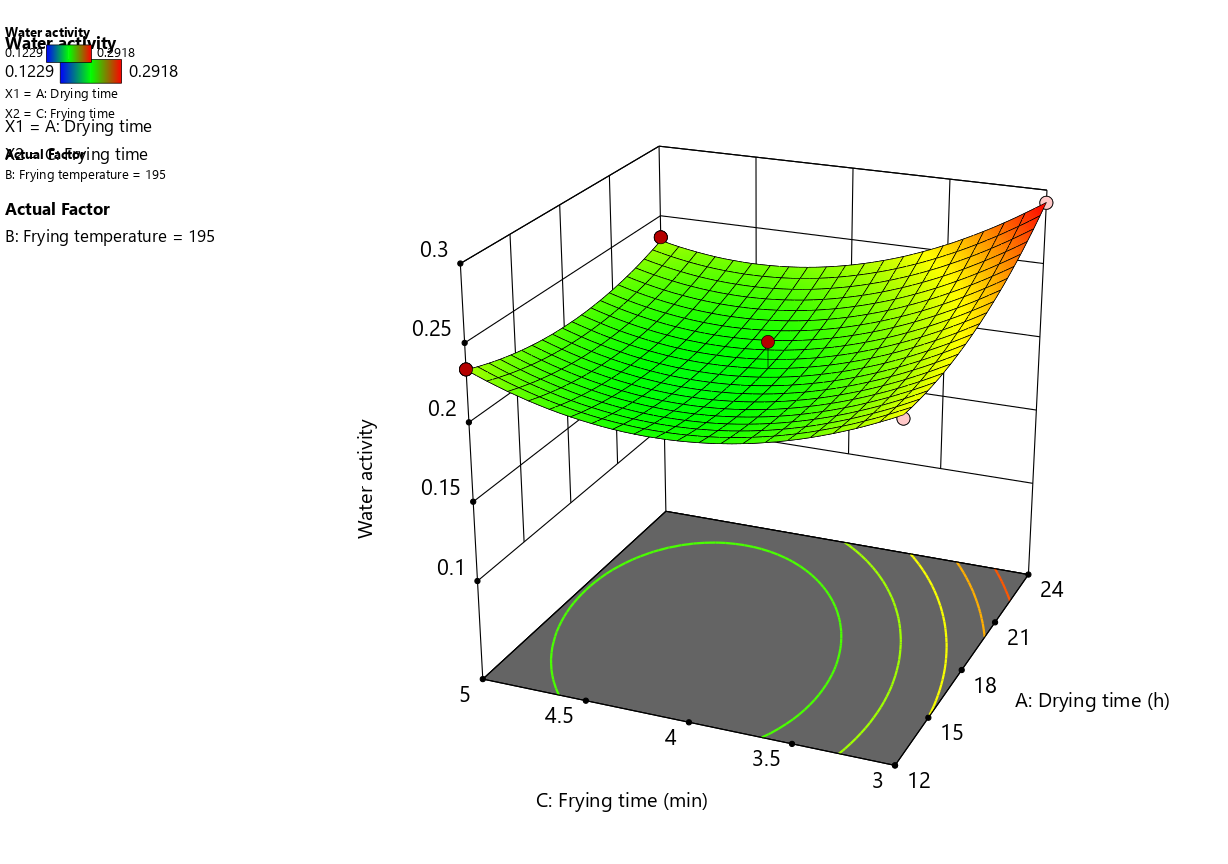 | 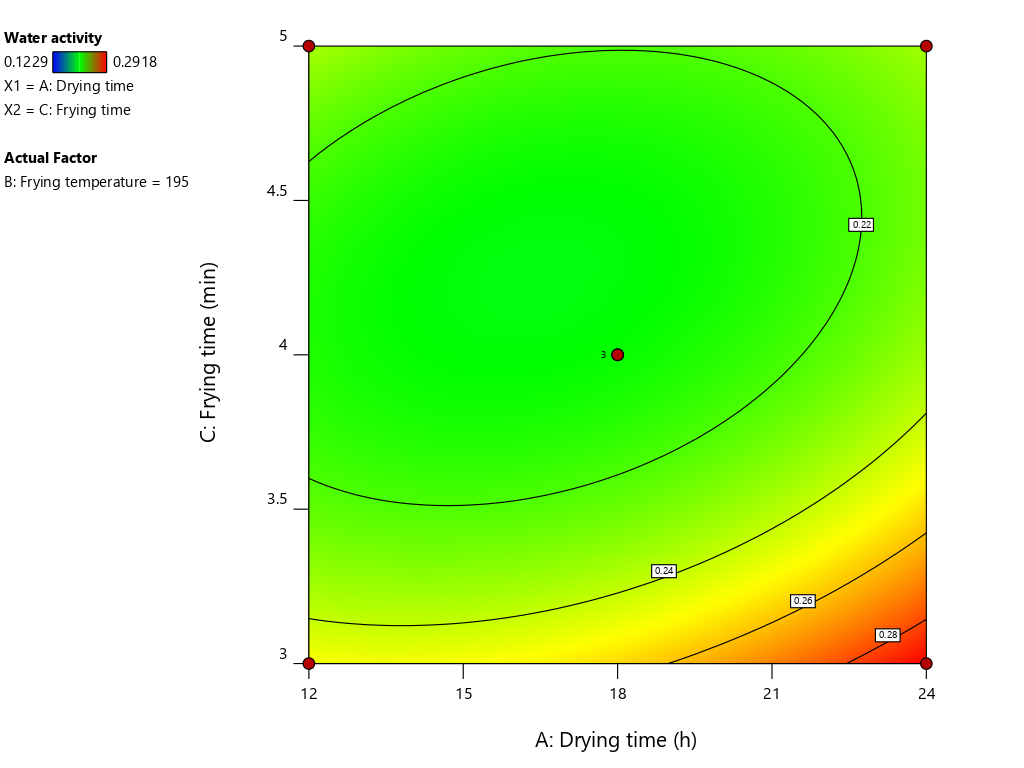 |
| 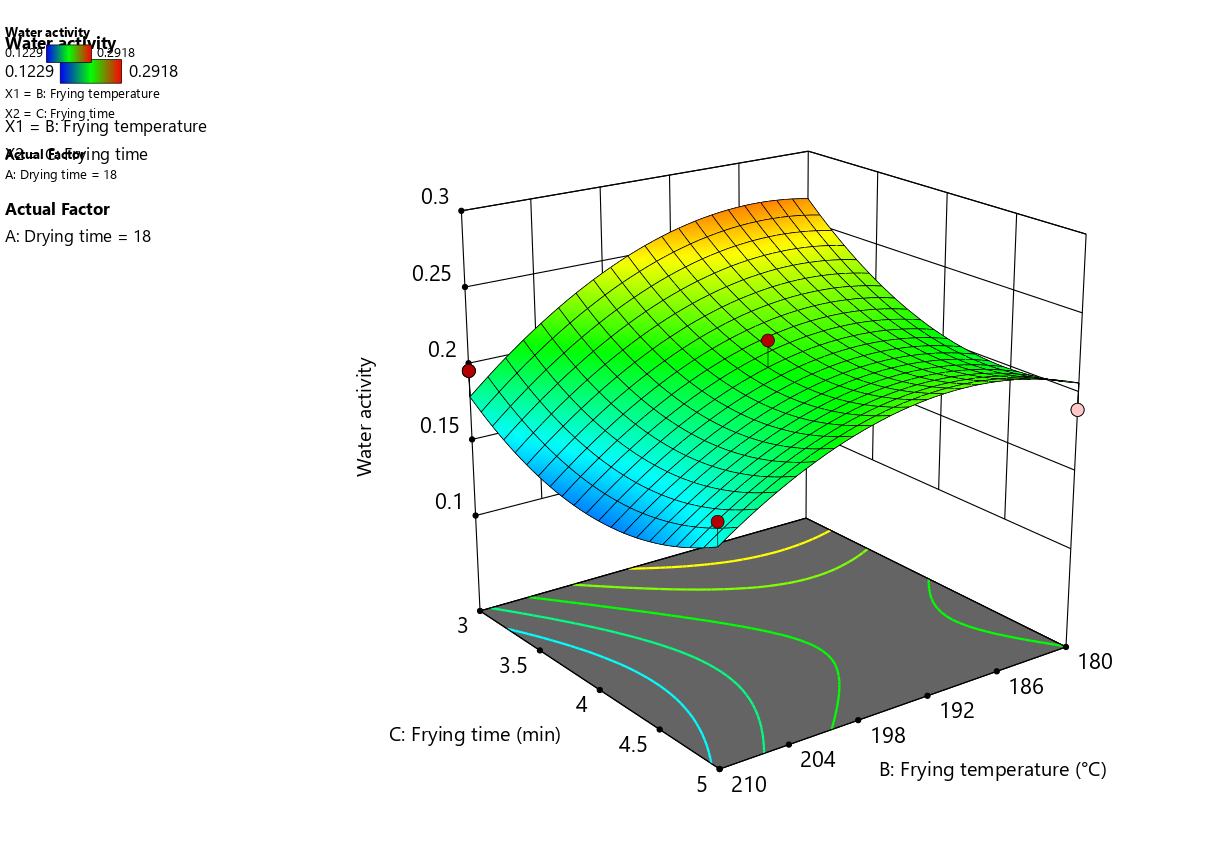 | 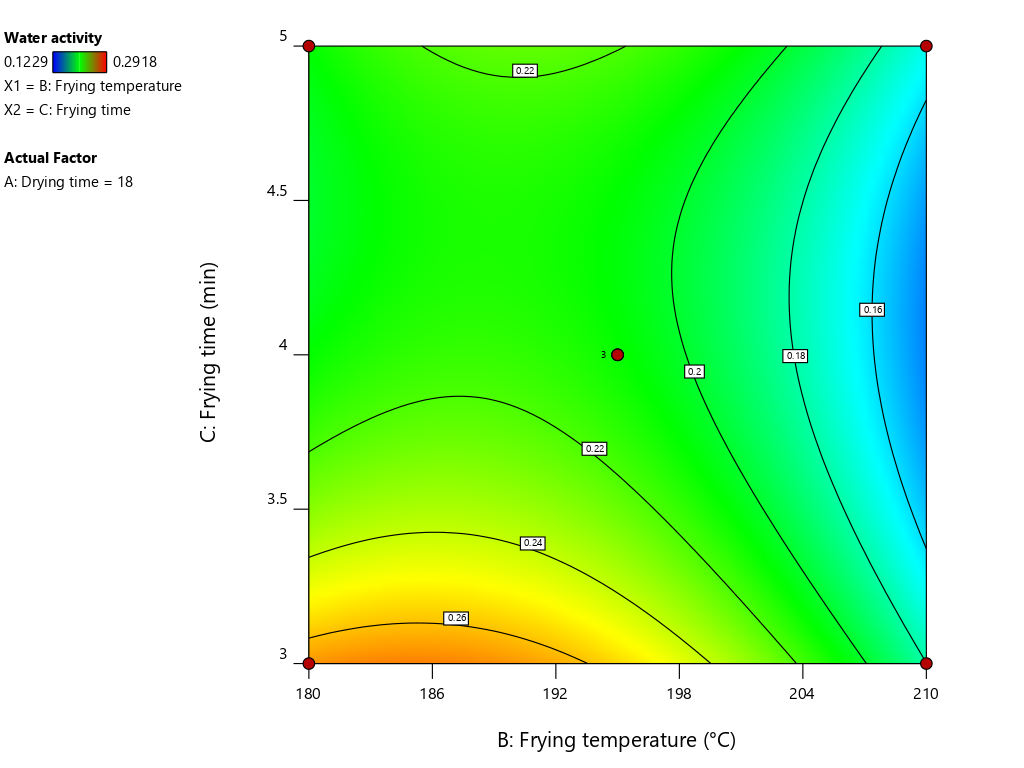 |

Appendix 1 Response surface plot for effect of different drying time, frying temperature, and frying time on water activity of fried pork rinds.

Appendix 2a Analysis of variance and regression for response surface model parameters affecting moisture content of fried pork rinds

| Source | Sum of squares | df | Mean square | F-value | p-value |  |
| --- | --- | --- | --- | --- | --- | --- |
| Model | 8.50 | 9 | 0.9447 | 5.69 | 0.0350 | significant |
| A-Drying time | 1.76 | 1 | 1.76 | 10.59 | 0.0226 |  |
| B-Frying temperature | 4.16 | 1 | 4.16 | 25.07 | 0.0041 |  |
| C-Frying time | 1.66 | 1 | 1.66 | 9.98 | 0.0251 |  |
| AB | 0.0462 | 1 | 0.0462 | 0.2785 | 0.6203 |  |
| AC | 0.3249 | 1 | 0.3249 | 1.96 | 0.2207 |  |
| BC | 0.0016 | 1 | 0.0016 | 0.0096 | 0.9256 |  |
| A² | 0.2700 | 1 | 0.2700 | 1.63 | 0.2582 |  |
| B² | 0.0205 | 1 | 0.0205 | 0.1237 | 0.7393 |  |
| C² | 0.2750 | 1 | 0.2750 | 1.66 | 0.2544 |  |
| Residual | 0.8299 | 5 | 0.1660 |  |  |  |
| Lack of fit | 0.5814 | 3 | 0.1938 | 1.56 | 0.4136 | not significant |
| Pure error | 0.2485 | 2 | 0.1242 |  |  |  |
| Cor total | 9.33 | 14 |  |  |  |  |
| R^2^ | 0.9111 |  |  |  |  |  |
| Adjusted R^2^ | 0.7510 |  |  |  |  |  |

The model F-value of 5.69 implies the model is significant. There is only a 3.50% chance that an F-value this large could occur due to noise.

P-values less than 0.0500 indicate model terms are significant.

The lack of fit F-value of 1.56 implies the lack of fit is not significant relative to the pure error. There is a 41.36% chance that a lack of fit F-value this large could occur due to noise.

Appendix 2b Analysis of variance and regression for response surface model parameters affecting oil content of fried pork rinds

| Source | Sum of squares | df | Mean square | F-value | p-value |  |
| --- | --- | --- | --- | --- | --- | --- |
| Model | 319.42 | 9 | 35.49 | 2.95 | 0.1230 | not significant |
| A-Drying time | 3.11 | 1 | 3.11 | 0.2588 | 0.6326 |  |
| B-Frying temperature | 2.71 | 1 | 2.71 | 0.2257 | 0.6548 |  |
| C-Frying time | 64.70 | 1 | 64.70 | 5.38 | 0.0681 |  |
| AB | 0.5041 | 1 | 0.5041 | 0.0419 | 0.8459 |  |
| AC | 5.31 | 1 | 5.31 | 0.4418 | 0.5357 |  |
| BC | 6.15 | 1 | 6.15 | 0.5114 | 0.5065 |  |
| A² | 7.48 | 1 | 7.48 | 0.6223 | 0.4659 |  |
| B² | 222.99 | 1 | 222.99 | 18.54 | 0.0077 |  |
| C² | 4.40 | 1 | 4.40 | 0.3656 | 0.5718 |  |
| Residual | 60.14 | 5 | 12.03 |  |  |  |
| Lack of fit | 59.88 | 3 | 19.96 | 153.65 | 0.0065 | significant |
| Pure error | 0.2598 | 2 | 0.1299 |  |  |  |
| Cor total | 379.55 | 14 |  |  |  |  |
| R^2^ | 0.8416 |  |  |  |  |  |
| Adjusted R^2^ | 0.5564 |  |  |  |  |  |

The model F-value of 2.95 implies the model is not significant relative to the noise. There is a 12.30% chance that an F-value this large could occur due to noise.

P-values less than 0.0500 indicate model terms are significant.

The lack of fit F-value of 153.65 implies the lack of fit is significant. There is only a 0.65% chance that a lack of fit F-value this large could occur due to noise.

Appendix 2c Analysis of variance and regression for response surface model parameters affecting puffing ratio of fried pork rinds

| Source | Sum of squares | df | Mean square | F-value | p-value |  |
| --- | --- | --- | --- | --- | --- | --- |
| Model | 2.282 × 10^5^ | 9 | 25353.38 | 1.74 | 0.2819 | not significant |
| A-Drying time | 95013.12 | 1 | 95013.12 | 6.51 | 0.0512 |  |
| B-Frying temperature | 23575.98 | 1 | 23575.98 | 1.61 | 0.2598 |  |
| C-Frying time | 1554.31 | 1 | 1554.31 | 0.1064 | 0.7575 |  |
| AB | 6852.53 | 1 | 6852.53 | 0.4692 | 0.5238 |  |
| AC | 6313.89 | 1 | 6313.89 | 0.4323 | 0.5399 |  |
| BC | 472.41 | 1 | 472.41 | 0.0323 | 0.8643 |  |
| A² | 32946.19 | 1 | 32946.19 | 2.26 | 0.1934 |  |
| B² | 62446.81 | 1 | 62446.81 | 4.28 | 0.0935 |  |
| C² | 1819.10 | 1 | 1819.10 | 0.1246 | 0.7385 |  |
| Residual | 73020.32 | 5 | 14604.06 |  |  |  |
| Lack of fit | 46357.73 | 3 | 15452.58 | 1.16 | 0.4942 | not significant |
| Pure error | 26662.59 | 2 | 13331.30 |  |  |  |
| Cor total | 3.012 × 10^5^ | 14 |  |  |  |  |
| R^2^ | 0.7576 |  |  |  |  |  |
| Adjusted R^2^ | 0.3212 |  |  |  |  |  |

The model F-value of 1.74 implies the model is not significant relative to the noise. There is a 28.19% chance that an F-value this large could occur due to noise.

P-values less than 0.0500 indicate model terms are significant.

The lack of fit F-value of 1.16 implies the lack of fit is not significant relative to the pure error. There is a 49.42% chance that a lack of fit F-value this large could occur due to noise.

Appendix 2d Analysis of variance and regression for response surface model parameters affecting breaking force of fried pork rinds

| Source | Sum of squares | df | Mean square | F-value | p-value |  |
| --- | --- | --- | --- | --- | --- | --- |
| Model | 2681.19 | 9 | 297.91 | 7.52 | 0.0194 | significant |
| A-Drying time | 634.18 | 1 | 634.18 | 16.00 | 0.0103 |  |
| B-Frying temperature | 777.64 | 1 | 777.64 | 19.62 | 0.0068 |  |
| C-Frying time | 178.98 | 1 | 178.98 | 4.52 | 0.0870 |  |
| AB | 87.45 | 1 | 87.45 | 2.21 | 0.1976 |  |
| AC | 49.85 | 1 | 49.85 | 1.26 | 0.3130 |  |
| BC | 309.92 | 1 | 309.92 | 7.82 | 0.0382 |  |
| A² | 4.65 | 1 | 4.65 | 0.1172 | 0.7460 |  |
| B² | 544.41 | 1 | 544.41 | 13.74 | 0.0139 |  |
| C² | 136.82 | 1 | 136.82 | 3.45 | 0.1223 |  |
| Residual | 198.18 | 5 | 39.64 |  |  |  |
| Lack of fit | 197.62 | 3 | 65.87 | 234.43 | 0.0043 | significant |
| Pure error | 0.5620 | 2 | 0.2810 |  |  |  |
| Cor total | 2879.37 | 14 |  |  |  |  |
| R^2^ | 0.9312 |  |  |  |  |  |
| Adjusted R^2^ | 0.8073 |  |  |  |  |  |

The model F-value of 7.52 implies the model is significant. There is only a 1.94% chance that an F-value this large could occur due to noise.

P-values less than 0.0500 indicate model terms are significant.

The lack of fit F-value of 234.43 implies the lack of fit is significant. There is only a 0.43% chance that a lack of fit F-value this large could occur due to noise.

Appendix 2e Analysis of variance and regression for response surface model parameters affecting water activity of fried pork rinds

| Source | Sum of squares | df | Mean square | F-value | p-value |  |
| --- | --- | --- | --- | --- | --- | --- |
| Model | 0.0214 | 9 | 0.0024 | 4.76 | 0.0503 | not significant |
| A-Drying time | 0.0009 | 1 | 0.0009 | 1.75 | 0.2428 |  |
| B-Frying temperature | 0.0080 | 1 | 0.0080 | 15.93 | 0.0104 |  |
| C-Frying time | 0.0025 | 1 | 0.0025 | 5.05 | 0.0746 |  |
| AB | 0.0005 | 1 | 0.0005 | 0.9233 | 0.3807 |  |
| AC | 0.0005 | 1 | 0.0005 | 0.9755 | 0.3687 |  |
| BC | 0.0006 | 1 | 0.0006 | 1.13 | 0.3361 |  |
| A² | 0.0008 | 1 | 0.0008 | 1.53 | 0.2704 |  |
| B² | 0.0039 | 1 | 0.0039 | 7.75 | 0.0387 |  |
| C² | 0.0033 | 1 | 0.0033 | 6.52 | 0.0511 |  |
| Residual | 0.0025 | 5 | 0.0005 |  |  |  |
| Lack of fit | 0.0020 | 3 | 0.0007 | 2.90 | 0.2666 | not significant |
| Pure error | 0.0005 | 2 | 0.0002 |  |  |  |
| Cor total | 0.0239 | 14 |  |  |  |  |
| R^2^ | 0.8954 |  |  |  |  |  |
| Adjusted R^2^ | 0.7072 |  |  |  |  |  |

The model F-value of 4.76 implies there is a 5.03% chance that an F-value this large could occur due to noise.

P-values less than 0.0500 indicate model terms are significant.

The lack of fit F-value of 2.90 implies the lack of fit is not significant relative to the pure error. There is a 26.66% chance that a lack of fit F-value this large could occur due to noise.

Appendix 2f Analysis of variance and regression for response surface model parameters affecting L* value of fried pork rinds

| Source | Sum of squares | df | Mean square | F-value | p-value |  |
| --- | --- | --- | --- | --- | --- | --- |
| Model | 129.95 | 9 | 14.44 | 2.52 | 0.1611 | not significant |
| A-Drying time | 5.87 | 1 | 5.87 | 1.02 | 0.3584 |  |
| B-Frying temperature | 0.1770 | 1 | 0.1770 | 0.0308 | 0.8675 |  |
| C-Frying time | 30.11 | 1 | 30.11 | 5.25 | 0.0706 |  |
| AB | 5.18 | 1 | 5.18 | 0.9019 | 0.3859 |  |
| AC | 20.25 | 1 | 20.25 | 3.53 | 0.1191 |  |
| BC | 2.37 | 1 | 2.37 | 0.4133 | 0.5486 |  |
| A² | 11.55 | 1 | 11.55 | 2.01 | 0.2152 |  |
| B² | 22.87 | 1 | 22.87 | 3.99 | 0.1024 |  |
| C² | 27.09 | 1 | 27.09 | 4.72 | 0.0819 |  |
| Residual | 28.69 | 5 | 5.74 |  |  |  |
| Lack of fit | 18.34 | 3 | 6.11 | 1.18 | 0.4892 | not significant |
| Pure error | 10.36 | 2 | 5.18 |  |  |  |
| Cor total | 158.64 | 14 |  |  |  |  |
| R^2^ | 0.8191 |  |  |  |  |  |
| Adjusted R^2^ | 0.4936 |  |  |  |  |  |

The model F-value of 2.52 implies the model is not significant relative to the noise. There is a 16.11% chance that an F-value this large could occur due to noise.

P-values less than 0.0500 indicate model terms are significant.

The lack of fit F-value of 1.18 implies the lack of fit is not significant relative to the pure error. There is a 48.92% chance that a lack of fit F-value this large could occur due to noise.

Appendix 2g Analysis of variance and regression for response surface model parameters affecting a* value of fried pork rinds

| Source | Sum of Squares | df | Mean Square | F-value | p-value |  |
| --- | --- | --- | --- | --- | --- | --- |
| Model | 16.47 | 9 | 1.83 | 15.61 | 0.0037 | significant |
| A-Drying time | 7.45 | 1 | 7.45 | 63.53 | 0.0005 |  |
| B-Frying temperature | 3.37 | 1 | 3.37 | 28.71 | 0.0030 |  |
| C-Frying time | 0.8778 | 1 | 0.8778 | 7.49 | 0.0410 |  |
| AB | 0.4489 | 1 | 0.4489 | 3.83 | 0.1078 |  |
| AC | 0.0100 | 1 | 0.0100 | 0.0853 | 0.7820 |  |
| BC | 0.1482 | 1 | 0.1482 | 1.26 | 0.3119 |  |
| A² | 1.37 | 1 | 1.37 | 11.70 | 0.0188 |  |
| B² | 2.01 | 1 | 2.01 | 17.11 | 0.0090 |  |
| C² | 0.6761 | 1 | 0.6761 | 5.77 | 0.0615 |  |
| Residual | 0.5863 | 5 | 0.1173 |  |  |  |
| Lack of fit | 0.2010 | 3 | 0.0670 | 0.3479 | 0.7992 | not significant |
| Pure error | 0.3853 | 2 | 0.1926 |  |  |  |
| Cor total | 17.05 | 14 |  |  |  |  |
| R^2^ | 0.9656 |  |  |  |  |  |
| Adjusted R^2^ | 0.9037 |  |  |  |  |  |

The model F-value of 15.61 implies the model is significant. There is only a 0.37% chance that an F-value this large could occur due to noise.

P-values less than 0.0500 indicate model terms are significant.

The lack of fit F-value of 0.35 implies the lack of fit is not significant relative to the pure error. There is a 79.92% chance that a lack of fit F-value this large could occur due to noise.

Appendix 2h Analysis of variance and regression for response surface model parameters affecting b* value of fried pork rinds

| Source | Sum of squares | df | Mean square | F-value | p-value |  |
| --- | --- | --- | --- | --- | --- | --- |
| Model | 126.70 | 9 | 14.08 | 4.79 | 0.0497 | significant |
| A-Drying time | 7.24 | 1 | 7.24 | 2.46 | 0.1774 |  |
| B-Frying temperature | 90.52 | 1 | 90.52 | 30.79 | 0.0026 |  |
| C-Frying time | 0.2048 | 1 | 0.2048 | 0.0697 | 0.8024 |  |
| AB | 0.4900 | 1 | 0.4900 | 0.1667 | 0.7000 |  |
| AC | 10.66 | 1 | 10.66 | 3.63 | 0.1153 |  |
| BC | 1.58 | 1 | 1.58 | 0.5357 | 0.4971 |  |
| A² | 6.98 | 1 | 6.98 | 2.37 | 0.1840 |  |
| B² | 6.05 | 1 | 6.05 | 2.06 | 0.2109 |  |
| C² | 5.43 | 1 | 5.43 | 1.85 | 0.2323 |  |
| Residual | 14.70 | 5 | 2.94 |  |  |  |
| Lack of fit | 12.57 | 3 | 4.19 | 3.92 | 0.2098 | not significant |
| Pure error | 2.14 | 2 | 1.07 |  |  |  |
| Cor total | 141.40 | 14 |  |  |  |  |
| R^2^ | 0.8960 |  |  |  |  |  |
| Adjusted R^2^ | 0.7089 |  |  |  |  |  |

The model F-value of 4.79 implies the model is significant. There is only a 4.97% chance that an F-value this large could occur due to noise.

P-values less than 0.0500 indicate model terms are significant.

The lack of fit F-value of 3.92 implies the lack of fit is not significant relative to the pure error. There is a 20.98% chance that a lack of fit F-value this large could occur due to noise.

Appendix 2i Analysis of variance and regression for response surface model parameters affecting b* value of fried pork rinds

| Source | Sum of Squares | df | Mean Square | F-value | p-value |  |
| --- | --- | --- | --- | --- | --- | --- |
| Model | 126.85 | 9 | 14.09 | 2.07 | 0.2191 | not significant |
| A-Drying time | 3.18 | 1 | 3.18 | 0.4660 | 0.5252 |  |
| B-Frying temperature | 8.90 | 1 | 8.90 | 1.31 | 0.3047 |  |
| C-Frying time | 27.23 | 1 | 27.23 | 4.00 | 0.1021 |  |
| AB | 3.08 | 1 | 3.08 | 0.4520 | 0.5312 |  |
| AC | 27.62 | 1 | 27.62 | 4.05 | 0.1002 |  |
| BC | 1.60 | 1 | 1.60 | 0.2348 | 0.6484 |  |
| A² | 6.12 | 1 | 6.12 | 0.8977 | 0.3869 |  |
| B² | 14.65 | 1 | 14.65 | 2.15 | 0.2024 |  |
| C² | 30.26 | 1 | 30.26 | 4.44 | 0.0889 |  |
| Residual | 34.07 | 5 | 6.81 |  |  |  |
| Lack of fit | 22.48 | 3 | 7.49 | 1.29 | 0.4641 | not significant |
| Pure error | 11.59 | 2 | 5.80 |  |  |  |
| Cor total | 160.92 | 14 |  |  |  |  |
| R^2^ | 0.7883 |  |  |  |  |  |
| Adjusted R^2^ | 0.4072 |  |  |  |  |  |

The model F-value of 2.07 implies the model is not significant relative to the noise. There is a 21.91% chance that an F-value this large could occur due to noise.

P-values less than 0.0500 indicate model terms are significant.

The lack of fit F-value of 1.29 implies the lack of fit is not significant relative to the pure error. There is a 46.41% chance that a lack of fit F-value this large could occur due to noise.

Appendix 3 Solutions of optimal conditions 1 (opt. 1) for response surface methodology of fried pork rinds.

| Number | Drying time  (h) | Frying temperature  (℃) | Frying time  (min) | Moisture content  (g/100 g wb) | Oil content  (g/100 g db) | Puffing ratio  (%) | Breaking force  (N) | Water activity | L* | a* | b* | Desirability |
| --- | --- | --- | --- | --- | --- | --- | --- | --- | --- | --- | --- | --- |
| 1 | 24.000 | 210.000 | 3.000 | 0.923 | 26.601 | 738.779 | 32.190 | 0.227 | 78.924 | -9.598 | 44.080 | 0.793 |
| 2 | 24.000 | 209.999 | 3.009 | 0.917 | 26.607 | 737.852 | 32.151 | 0.226 | 78.848 | -9.589 | 44.041 | 0.793 |
| 3 | 23.956 | 210.000 | 3.000 | 0.924 | 26.624 | 739.372 | 32.231 | 0.226 | 78.918 | -9.597 | 44.057 | 0.793 |
| 4 | 23.957 | 210.000 | 3.010 | 0.917 | 26.629 | 738.302 | 32.186 | 0.226 | 78.833 | -9.587 | 44.014 | 0.793 |
| 5 | 23.999 | 210.000 | 3.031 | 0.902 | 26.621 | 735.456 | 32.056 | 0.225 | 78.653 | -9.566 | 43.941 | 0.793 |
| 6 | 24.000 | 209.771 | 3.000 | 0.935 | 26.804 | 742.351 | 32.035 | 0.228 | 78.990 | -9.628 | 43.985 | 0.793 |
| 7 | 24.000 | 209.999 | 3.043 | 0.893 | 26.629 | 734.124 | 32.003 | 0.224 | 78.547 | -9.554 | 43.886 | 0.793 |
| 8 | 24.000 | 209.742 | 3.000 | 0.936 | 26.829 | 742.803 | 32.016 | 0.229 | 78.999 | -9.632 | 43.973 | 0.793 |
| 9 | 23.848 | 209.999 | 3.000 | 0.926 | 26.678 | 740.802 | 32.330 | 0.225 | 78.905 | -9.594 | 44.002 | 0.793 |
| 10 | 23.812 | 210.000 | 3.014 | 0.917 | 26.702 | 739.753 | 32.300 | 0.224 | 78.778 | -9.579 | 43.923 | 0.793 |
| 11 | 23.821 | 209.993 | 3.050 | 0.892 | 26.727 | 735.906 | 32.132 | 0.221 | 78.470 | -9.543 | 43.765 | 0.792 |
| 12 | 23.999 | 209.435 | 3.008 | 0.947 | 27.098 | 746.683 | 31.784 | 0.230 | 79.021 | -9.664 | 43.814 | 0.792 |
| 13 | 23.718 | 210.000 | 3.041 | 0.900 | 26.764 | 738.065 | 32.268 | 0.221 | 78.533 | -9.548 | 43.757 | 0.792 |
| 14 | 24.000 | 209.404 | 3.000 | 0.954 | 27.120 | 747.965 | 31.800 | 0.231 | 79.097 | -9.675 | 43.835 | 0.792 |
| 15 | 23.588 | 209.999 | 3.001 | 0.931 | 26.803 | 743.850 | 32.570 | 0.223 | 78.860 | -9.585 | 43.871 | 0.792 |
| 16 | 23.713 | 210.000 | 3.084 | 0.871 | 26.794 | 733.669 | 32.103 | 0.218 | 78.174 | -9.506 | 43.572 | 0.792 |
| 17 | 23.464 | 209.999 | 3.000 | 0.934 | 26.860 | 745.317 | 32.692 | 0.222 | 78.846 | -9.581 | 43.815 | 0.791 |
| 18 | 23.849 | 209.212 | 3.000 | 0.967 | 27.357 | 752.767 | 31.828 | 0.230 | 79.134 | -9.697 | 43.681 | 0.791 |
| 19 | 24.000 | 208.984 | 3.043 | 0.946 | 27.502 | 749.563 | 31.336 | 0.230 | 78.836 | -9.684 | 43.474 | 0.791 |
| 20 | 24.000 | 208.836 | 3.000 | 0.983 | 27.592 | 756.332 | 31.464 | 0.234 | 79.255 | -9.747 | 43.606 | 0.791 |
| 21 | 23.366 | 210.000 | 3.110 | 0.860 | 26.968 | 735.065 | 32.314 | 0.213 | 77.913 | -9.468 | 43.304 | 0.790 |
| 22 | 24.000 | 208.679 | 3.009 | 0.985 | 27.725 | 757.577 | 31.328 | 0.235 | 79.214 | -9.757 | 43.502 | 0.790 |
| 23 | 24.000 | 209.046 | 3.127 | 0.888 | 27.520 | 739.882 | 31.031 | 0.224 | 78.111 | -9.593 | 43.140 | 0.790 |
| 24 | 23.949 | 210.000 | 3.191 | 0.800 | 26.775 | 719.508 | 31.579 | 0.213 | 77.330 | -9.413 | 43.236 | 0.790 |
| 25 | 23.134 | 210.000 | 3.169 | 0.827 | 27.107 | 731.823 | 32.326 | 0.207 | 77.427 | -9.404 | 42.975 | 0.789 |
| 26 | 24.000 | 207.913 | 3.000 | 1.030 | 28.310 | 769.139 | 30.993 | 0.240 | 79.496 | -9.859 | 43.242 | 0.787 |
| 27 | 24.000 | 207.654 | 3.131 | 0.956 | 28.621 | 758.851 | 30.200 | 0.232 | 78.430 | -9.756 | 42.580 | 0.785 |
| 28 | 22.371 | 210.000 | 3.019 | 0.957 | 27.322 | 752.069 | 33.665 | 0.210 | 78.443 | -9.498 | 43.266 | 0.784 |
| 29 | 24.000 | 207.166 | 3.090 | 1.007 | 28.931 | 769.328 | 30.165 | 0.237 | 78.889 | -9.852 | 42.566 | 0.784 |
| 30 | 22.449 | 210.000 | 3.377 | 0.717 | 27.546 | 719.543 | 32.489 | 0.189 | 75.897 | -9.195 | 42.001 | 0.781 |
| 31 | 22.190 | 210.000 | 3.446 | 0.685 | 27.702 | 715.957 | 32.652 | 0.183 | 75.448 | -9.129 | 41.717 | 0.778 |
| 32 | 24.000 | 209.999 | 3.735 | 0.559 | 27.614 | 670.686 | 32.120 | 0.187 | 73.890 | -9.061 | 41.408 | 0.757 |
| 33 | 24.000 | 207.794 | 3.764 | 0.662 | 29.612 | 701.704 | 30.009 | 0.198 | 74.270 | -9.303 | 40.518 | 0.755 |
| 34 | 24.000 | 202.306 | 3.000 | 1.305 | 31.412 | 825.786 | 30.102 | 0.268 | 80.557 | -10.420 | 41.236 | 0.752 |

Appendix 4 Solutions of optimal conditions 2 (opt. 2) for response surface methodology of fried pork rinds.

| Number | Drying time  (h) | Frying temperature  (℃) | Frying time  (min) | Moisture content  (g/100 g wb) | Oil content  (g/100 g db) | Puffing ratio  (%) | Breaking force  (N) | Water activity | L* | a* | b* | Desirability |
| --- | --- | --- | --- | --- | --- | --- | --- | --- | --- | --- | --- | --- |
| 1 | 22.349 | 197.245 | 3.832 | 1.140 | 35.000 | 792.841 | 28.283 | 0.224 | 75.348 | -10.048 | 36.799 | 0.890 |
| 2 | 24.000 | 188.058 | 4.399 | 1.428 | 35.000 | 719.496 | 30.611 | 0.227 | 71.486 | -9.900 | 35.074 | 0.815 |

Appendix 5a Predicted and experimental result of optimal conditions for response surface methodology of fried pork rinds: (a) Optimal condition 1 and (b) Optimal condition 2.

(a)

| Optimal condition 1 (opt. 1):  drying time: 24h; frying temperature: 210℃ ; frying time: 3 min. | | | | |
| --- | --- | --- | --- | --- |
| Response | Predicted result | Experimental result | 95% confidence interval | 95% prediction interval |
| Moisture content (g/100 g wb) | 0.92 ± 0.41 | 1.26 ± 0.23 | (-0.31, 2.16) | (-0.71, 2.54) |
| Oil content (g/100 g db) | 26.60 ± 3.47 | 31.52 ± 2.46 | (16.07, 37.13) | (12.80, 40.40) |
| Puffing ratio (%) | 738.78 ± 120.85 | 423.58 ± 42.61 | (371.76, 1105.79) | (257.95, 1219.61) |
| Breaking force (N) | 32.191 ± 6.296 | 49.871 ± 2.589 | (13.070, 51.311) | (7.141, 57.240) |
| Water activity (a_w_) | 0.2269 ± 0.0224 | 0.1612 ± 0.0178 | (0.1589, 0.2949) | (0.1379, 0.3159) |
| L^*^ | 78.92 ± 2.40 | 76.31 ± 1.06 | (71.65, 86.20) | (69.39, 88.45) |
| a^*^ | -9.60 ± 0.34 | -8.61 ± 0.35 | (-10.64, -8.56) | (-10.96, -8.24) |
| b^*^ | 44.08 ± 1.71 | 42.72 ± 1.41 | (38.87, 49.29) | (37.26, 50.90) |

Expressed as mean ± standard deviation (n=3).

Appendix 5b Predicted and experimental result of optimal conditions for response surface methodology of fried pork rinds: (a) Optimal condition 1 and (b) Optimal condition 2.

(b)

| Optimal condition 2 (opt. 2):  drying time: 22.35h; frying temperature: 197℃ ; frying time: 3.8 min. | | | | |
| --- | --- | --- | --- | --- |
| Response | Predicted result | Experimental result | 95% confidence interval | 95% prediction interval |
| Moisture content (g/100 g wb) | 1.16 ± 0.41 | 0.96 ± 0.10 | (0.59, 1.73) | (-0.03, 2.35) |
| Oil content (g/100 g db) | 34.91 ± 3.47 | 40.37 ± 2.94 | (30.07, 39.77) | (24.77, 45.07) |
| Puffing ratio (%) | 794.69 ± 120.85 | 805.51 ± 60.11 | (625.62, 963.76) | (441.02, 1148.37) |
| Breaking force (N) | 28.459 ± 6.296 | 25.022 ± 3.279 | (19.651, 37.270) | (10.034, 46.884) |
| Water activity (a_w_) | 0.2261 ± 0.0224 | 0.1924 ± 0.0130 | (0.1948, 0.2574) | (0.1606, 0.2915) |
| L^*^ | 75.49 ± 2.40 | 71.14 ± 3.86 | (72.14, 78.84) | (68.48, 82.50) |
| a^*^ | -10.07 ± 0.34 | -9.32 ± 0.36 | (-10.55, -9.59) | (-11.08, -8.80) |
| b^*^ | 36.79 ±1.71 | 35.23 ± 1.87 | (34.39, 39.19) | (31.77, 41.81) |

Expressed as mean ± standard deviation (n=3).
